# Rules for robots, and why medical AI breaks them

**DOI:** 10.1093/jlb/lsad001

**Published:** 2023-02-16

**Authors:** Barbara J Evans

**Affiliations:** University of Florida Levin College of Law, Gainesville, FL, USA; University of Florida Wertheim College of Engineering, Gainesville, FL, USA

**Keywords:** White House Blueprint for an AI Bill of Rights, clinical decision support (CDS) software, medical AI, health data privacy, bias, equitable AI

## Abstract

This article critiques the quest to state general rules to protect human rights against AI/ML computational tools. The White House Blueprint for an AI Bill of Rights was a recent attempt that fails in ways this article explores. There are limits to how far ethicolegal analysis can go in abstracting AI/ML tools, as a category, from the specific contexts where AI tools are deployed. Health technology offers a good example of this principle. The salient dilemma with AI/ML medical software is that privacy policy has the potential to undermine distributional justice, forcing a choice between two competing visions of privacy protection. The first, stressing individual consent, won favor among bioethicists, information privacy theorists, and policymakers after 1970 but displays an ominous potential to bias AI training data in ways that promote health care inequities. The alternative, an older duty-based approach from medical privacy law aligns with a broader critique of how late-20th-century American law and ethics endorsed atomistic autonomy as the highest moral good, neglecting principles of caring, social interdependency, justice, and equity. Disregarding the context of such choices can produce suboptimal policies when - as in medicine and many other contexts - the use of personal data has high social value.

## I. INTRODUCTION

The White House Office of Science and Technology Policy published a Blueprint for an AI Bill of Rights in October 2022.^1^ The quest to codify rights of humans as against smart machinery dates at least as far back as Isaac Asimov’s three laws for robots in 1942.^2^ It feels more pressing as smart machines crawl out of science fiction and into everyday life driving cars, serving ads for products an algorithm inferred you might want, screening job applicants, nixing people’s eligibility for home loans and parole, diagnosing whether a skin lesion is cancer, and advising the doctor how to treat it if it is.^3^ Carlos Ignacio Gutierrez and Gary Marchant recently counted 634 soft law programs worldwide striving to enunciate rules to protect the interests of humans in the age of AI.^4^

The White House’s Bill of Rights analogy presumes that a single, overarching set of norms can protect people’s interests in all contexts where artificial intelligence/machine learning (AI/ML) tools are used. This article questions that view, using an example drawn from medical AI as a proxy for a larger phenomenon, which is that many uses of AI tools raise context-specific legal and ethical concerns for which general rules are poorly tailored. Inspired by Helen Nissenbaum’s influential insight that privacy norms are contextual,^5^ this article suggests more broadly that *all* rules for robots and AI systems—not just the privacy rules—may need to be contextually informed and context-appropriate.

The love of general rules may be a uniquely human failing and, if so, it may prove to be the tragic flaw that dooms the struggle to protect human rights against smart robots and AI systems. Complexity and diversity are hard for humans. The human mind can only balance several factors—perhaps two to five—simultaneously. There is an inherent ‘mismatch between the mathematical optimization in high-dimensionality characteristic of machine learning and the [more limited] demands of human-scale reasoning.^6^’ Machine brains can weigh thousands of factors at once and cope with diversity and nuance unemotionally. If nuanced, context-appropriate rules are what humans need to survive against smart machines, then humans may lose, because humans are saddled with low-dimensionality, two- to five-factoral minds apt to compress complex realities into simplistic lists of general, all-encompassing rules, as the White House Blueprint for an AI Bill of Rights just did.

AI/ML clinical decision support (CDS) tools—the example this article explores—are a category of medical software providing ‘knowledge and person-specific information, intelligently filtered or presented at appropriate times, to enhance health and health care.^7^’ CDS tools combine information about an individual patient with various sources of general medical knowledge to develop patient-specific diagnostic or treatment recommendations.^8^ This general medical knowledge can include insights from peer-reviewed literature, practice guidelines, and FDA-approved drug labeling, but in the case of AI/ML CDS tools, it often include inferences generated internally by an AI/ML algorithm processing clinical health data drawn from many other patients treated in the past.^9^

AI/ML CDS tools are designed to assist (not replace) health care professionals in traditional health care settings such as clinics, hospitals, and nursing homes.^10^ They represent a large, important category of medical AI, rather like a flagship in the fleet now sailing into the future of AI-enabled clinical health care. The clinical use context distinguishes AI/ML CDS tools from consumer-facing health apps and health-inflected AI tools intended for use by laypeople,^11^ and from AI algorithms imbedded in traditional medical devices (such as software processing mammograms to highlight areas suspicious for disease).^12^ AI/ML CDS tools infer what might be wrong with a patient and how best to treat it by comparing information about that patient to lessons gleaned from many other people’s prior health care experiences. ^13^ They thirst for data (more data, better-quality data, more diverse and inclusive data reflecting all of us) and raise privacy concerns. They are already part of the workflow at many health care facilities, recommending diagnoses and treatments to health professionals and influencing patients’ care, often without the patients knowing AI is running in the background, suctioning their data,^14^ and guiding important decisions about their health.^15^

The contexts where AI tools are employed present distinct challenges and opportunities. These can overwhelm attempts to state one general set of rules for AI used in large-scale data collection and processing in modern surveillance societies.^16^ Part I surveys rules proposed for robotic and AI systems generally and argues that AI-enabled health care may require different rules, including different privacy rules, to protect individual rights while promoting social justice.

In making this argument, this article is aware that calls for medical exceptionalism often prove spurious. Policymakers still struggle to move past a recent error of this sort, genetic exceptionalism, which was the belief that ‘genetic tests should be treated differently from other laboratory tests for oversight purposes.’^17^ It sprang from deliberations of a federal task force working in 1991, just as the Human Genome Project was starting.^18^ Scholars feared the genome ‘holds dark, implacable secrets’ and ‘toxic knowledge’ that might overwhelm genetically unschooled users and require special regulatory guardrails.^19^ As scientists learned more about the uses (and the limits) of genetic tests, the case for exceptionalism grew weaker.^20^ Genetic tests, it turned out, were not all that different from many other diagnostic tests used in clinical and other settings. Medical AI may simply be the latest new thing that ‘holds dark, implacable secrets’ and ‘toxic knowledge’ in the minds of scholars hoping for something exceptional to write about.

With genetic exceptionalism as a cautionary example, proponents of exceptionalism should state what, precisely, warrants special treatment. Part I tries to do that for medical AI. If medical AI does, in fact, warrant special rules, this creates a jurisdictional quandary. Pervasive digital surveillance has blurred the line between medical and non-medical software. AI tools operating outside clinical health care settings can spot patterns in non-medical data and draw inferences about a person’s health. ^21^ If medical AI deserves special rules, should they apply to all AI that produces or processes health-related data, or just to AI intended for use in clinical health care? Part II argues that rules for AI should be tailored to the use context, rather than the data content, and suggests an intent-based approach for distinguishing which software must answer to special rules.

Any context-specific analysis—like this one—is only as generalizable as the context for which it was designed. Medical AI serves in this article as a metaphor, representative or symbolic of something else: that there may be limits to how far legal and ethical analysis can abstract AI, as a category, from the myriad use contexts in which AI is deployed in modern societies. General rule sets—‘carefully-delineated statements as to the thin common denominator of the wide legal terrain covered by the wholesale category’ of AI—can be enormously clarifying as long as we stay mindful that ‘the correct level of abstraction is a function of the purpose of our inquiry’.^22^ A crucial step when assessing social impacts of AI is to distinguish whether the problem is the AI or other, preexisting conditions of the environments where AI is in use.

## II. DIFFERENT RULES FOR DIFFERENT ROBOTS

To identify candidate rules for AI tools, this discussion takes as its signposts the ‘basic ethical principles’ set out in the 1979 Belmont Report, a foundational work in American medical research ethics.^23^ It supplements these principles with three influential statements of ‘rules for robots’ from legal and popular literature: those of Isaac Asimov, Jack Balkin, and Frank Pasquale.^24^ Their compact rule sets brim with insights and express shared truths and fears about human relations with smart machines and algorithms. After surveying the rules, this Part explores whether rules proposed for robots in general are well tailored for the special case of AI/ML CDS tools in clinical health care. Several of the rules do seem relevant. More troubling are a couple of the rules with question marks hanging over whether CDS tools comply with them or, indeed, whether they are even the right rules for medical software at all.

### II.A. From Asimov’s Rules for Robots to the Belmont Report: The Rise of Autonomy and Consent Norms

Isaac Asimov’s three laws for robots state broad ethical principles: (i) ‘a robot may not injure a human being or, through inaction, allow a human being to come to harm’; (ii) ‘a robot must obey orders given to it by human beings except where such orders would conflict with the First Law’; (iii) ‘a robot must protect its own existence as long as such protection does not conflict with the First or Second Laws’.^25^

Asimov’s laws arose in the same climate of mid-20th-century postwar liberalism—with its commitments to individual liberty, human rights, consent, and fairness—that also fueled the American bioethics movement in the late 1950s through 1970s.^26^ It is thus not surprising that Asimov’s laws resemble the ‘basic ethical principles’ of the Belmont Report, which inspired over 40 years of subsequent bioethical thought about ethical treatment of people volunteering for biomedical and behavioral research.^27^ The Belmont principles are ‘respect for persons’, ‘beneficence’ (which encompasses its do-no-harm twin, nonmaleficence), and ‘justice’.^28^

Like the Belmont principles, Asimov’s First and Second Laws emphasize robotic respect for persons and beneficence/nonmaleficence toward human beings. Asimov’s Second Law requires robots to obey human orders: that is, to act with human consent. His Third Law elevates these principles of respect for humans, beneficence/non-maleficence toward them, and human consent above other considerations, including the robot’s own survival. Justice has a minor, supporting role if robots are forced under Clause 2 of Asimov’s Second Law to adjudicate whether a human’s command contravenes the First Law against injuring human beings.

A similar skew emerged in American bioethics after the Belmont Report. Several senior scholars in the field liken bioethics to ‘a lopsided table with four legs’ in which ‘autonomy trumps other contenders’—that is, the other Belmont principles of beneficence, nonmaleficence, and justice.^29^ This skew flows from a choice about what ‘respect for persons’ entails. Post-Belmont bioethics embraced an atomistic notion of personhood, based on an ‘assumption of human separateness, where freedom is fundamental’ and ‘autonomy exercises radical self-direction’.^30^ If personhood is viewed this way, then ‘respect for someone as a person, as a chooser’ requires a strong norm of informed consent.^31^ After the Belmont Report, American bioethics embraced this view, consecrating individual consent as its emblem of respect for persons, ‘elevat[ing] the principles of autonomy and self-determination above other competing values in the hierarchy of ethical goods, such as beneficence, justice, dignity, and equality’.^32^

This choice was, in many ways, historically contingent. ‘The cultural preoccupation with individual autonomy is a distinctly post-World War II social phenomenon’ and ‘[c]ontemporary American bioethics developed in that milieu’.^33^ It was not, however, the only choice ethicists could have made. An alternative, social (relational) concept of personhood portrays individuals as fundamentally social, deriving identity from professional and personal relationships and ‘an array of competing communal principles that both identify the self and empower it to act’.^34^ This is the view, recently gaining ground in popular American culture, that identity rests not just on rugged independence but also on each person’s connections within a web of class, gender, racial, socioeconomic, and other affiliations. Proponents of this social-self school of thought, had they exerted greater influence on post-Belmont bioethics, might have set it on a path stressing other ethical principles: beneficence, justice, and health equity.

Instead, as things stand, the focus on consent subordinates the principle of beneficence to a degree that makes it hard, four decades later, to recall what ‘beneficence’ meant at the time it became the second Belmont principle.^35^ The principle of beneficence was not a mere platitude exhorting people to be kind but referred to an ‘an older ethic of physician beneficence’ that stressed physicians’ duties to ‘act as entrusted fiduciaries’ in the context of the social relationship between the physician and patient.^36^ Justice also takes a backseat to autonomy and consent: ‘The major moral and conceptual problems about informed consent are not justice-based and do not directly confront issues of social justice’.^37^

Exposed to decades of this brand of bioethical thinking, many Americans cited autonomy in refusing to comply with public health measures to protect others during the recent pandemic, and many feel ‘privacy is something to be protected at the discretion of the individual to whom the information relates’.^38^ Daniel Solove paraphrases modern ‘control-over-information’ privacy theory as ‘we protect all information over which individuals want to retain control’.^39^ This theory calls for individuals to consent before secondary uses of their identifiable health information, even if other people might have valid moral claims to the same information and even if the secondary uses would advance the wellbeing of other people, society in general, and the person the data describe.

The belief that data use should require consent is so widely held today that younger readers may be unaware of how recently it arose. The Belmont report focused mainly on clinical research but sparked a debate about whether its principles should also apply to informational research that uses people’s health data.^40^ A Privacy Protection Study Commission (PPSC) created by the Privacy Act of 1974 noticed that, as of 1977, medical researchers were routinely using patients’ clinical health records in research without consent.^41^ The National Commission for the Protection of Human Subjects of Biomedical and Behavioral Research confirmed that, as of 1978, consent was not the norm in informational studies ‘based exclusively upon existing records, data or materials [e.g., biospecimens] gathered for other purposes’.^42^ The PPSC recommended that people should be asked for consent before researchers use their identifiable health information.^43^

The postwar generation of bioethicists coming of age as scholars in the late 1970s embraced this norm of informed consent for research uses of personal data.^44^ Equivalent notice-and-consent norms later gained favor in the ‘Information Privacy Law Project’ examining data privacy in broader (non-medical) social and economic contexts.^45^ The concept of ‘privacy as a personal right to control the use of one’s data’ enjoyed ‘staggering’ consensus 20 years ago and was the ‘leading paradigm on the Internet and in the real, or off-line world’.^46^

Even 20 years ago, there were critics of control-over-information theory, which equates privacy with individual consent rights. Anita Allen noted that people who consent to share deeply personal information exercise control over the information, but the act of consenting entails losing a bit of what most people commonly view as privacy.^47^ More recently, it emerged that individuals’ privacy is interdependent. Others who consent to share information about themselves may inadvertently reveal facts about you, as when another partygoer’s selfie displays you passed out drunk in the background.^48^ Initially, this interdependency drew scholarly interest only in specific contexts, for example, when genetic testing reveals facts about family members^49^ or when research involving Indigenous people allows stigmatizing statistical inferences about all members of small tribal communities.^50^ In truth, the problem is more general. Privacy interdependency potentially affects everybody in a world of large-scale, generalizable data analytics. If people who are representative of you consent to be studied, a study can reveal facts about you even when you opt out.^51^ Privacy is not simply a function of who has access to your input data, which was how control-over-information theory conceived it. Privacy also depends on what can be inferred about you. You ultimately cannot control what other people infer.

In modern information economies, privacy loss is in part systemic, an inevitable consequence of the kinds of data analytics modern societies choose to pursue. The individual’s choice to contribute or withhold personal input data from those analyses will not necessarily prevent unwanted personal inferences from being drawn. Just as de-identification gradually loses its power to protect privacy as algorithms grow adept at inferring the stripped-off identifiers,^52^ so consent loses its power to protect privacy as algorithms grow less biased, more generalizable, better able to draw inferences about the non-consenters who opt out.

Despite these doubts about consent as a privacy protection, consent rights remain popular with the public and with many bioethicists and policymakers, who may pragmatically view consent rules as simpler and less costly than designing computer systems that would shield individuals from unwanted revelation of personal facts (whether by disclosure or by inference). The White House’s Blueprint for an AI Bill of Rights shrugs off modern concerns that consent is unable to bear the weight of protecting privacy. It recommends a notice-and-consent privacy scheme in which ‘designers, developers, and deployers of automated systems’ must ‘seek your permission’ to use data in an AI system.^53^ This aligns the Blueprint with the control-over-information theory that emerged after the Belmont report. Control-over-information theory is discredited, but it is far from dead.

The Belmont principles map Isaac Asimov’s rules for robots onto a set of rules for the humans who design and operate the robots and AI/ML software. The mapping is strikingly close for data privacy norms. Asimov’s rules require a robot to respect and protect humans, act with their consent, and destroy itself if it cannot do both. The Belmont principles call on biomedical researchers to respect persons who contribute data and biospecimens to research and to act with their consent. All that seems missing is an imperative for biomedical researchers to sacrifice their research mission, if necessary, to uphold consent norms. This, in fact, is not missing and is implicit in statements like this one by Franklin Miller of the National Institutes of Health’s Department of Bioethics:

The facts that historically much valuable population-based observational [informational] research has been conducted without informed consent, that obtaining consent would often make such research impossible to conduct, and that selection biases associated with soliciting consent may compromise its scientific validity do not in themselves constitute valid ethical reasons for waiving a requirement of informed consent.^54^

This statement applies Asimov’s Third Law to the facts of informational research. The ‘robot’ (in this analogy, informational research that has high social value) must obey Asimov’s Second Law (to follow orders/act with consent), even if doing so destroys the ‘robot’ itself—that is, even if requiring consent undermines the scientific validity of results. By this view, the moral imperative to obtain consent is so strong that science must sacrifice its own validity, if necessary, to uphold the norm of informed consent.

### II.B. The Ethic of Beneficence in a Control-over-Information World

Like the Belmont report, Jack Balkin and Frank Pasquale aim their rules for robots not at the robots, but at the humans who program them, operate them, use them, and ‘allow themselves to be governed by them’.^55^ Their rules address broader duties of users and controllers of AI systems, going well beyond privacy and the ethics of data use.^56^ This section discusses Prof. Balkin’s first rule, which relates to privacy and data ethics and defers their other rules to the next section.^57^

Prof. Balkin’s three laws would, first, require humans who are responsible for AI/ML algorithms to act as ‘information fiduciaries’ of their clients, customers, and end-users to whose data they have access.^58^ Second, responsible parties would have ‘public duties’ to uninvolved third parties—ie affected persons with whom they are not in privity of contract.^59^ Third, the responsible parties would have a duty not to externalize costs and harms of their operations.^60^

Absent from Prof. Balkin’s rules are the consent norms that figure so prominently in Asimov’s rules and in post-Belmont data ethics. In other work, he challenges scholars to ‘shift the focus’ in discussions of privacy ‘from the kind of *information* to the kinds of *relationships*—relationships of trust and confidence—that governments may regulate in the interests of privacy’.^61^ His ‘central point is that certain kinds of information constitute matters of private concern not because of their *content*, but because of the *social relationships* that produce them’.^62^ His first rule de-centers consent as a privacy protection and instead calls for placing those who handle people’s personal information under strong duties to be careful with it.^63^

This approach aligns with the relational view of personhood that, according to Alfred Tauber, was the road not taken by American bioethicists in the early years after the Belmont Report.^64^ Prof. Balkin’s first rule reinstates the older ‘ethic of beneficence’/‘ethic of care’—the second Belmont principle—that imposed a ‘primary ethical obligation’ for data handlers ‘to be responsible’ for protecting the privacy of patients whose data they receive.^65^

After the Belmont Report, many bioethicists and information privacy scholars converged on control-over-information theory, which ‘conceives of privacy as a personal right to control the use of one’s data’ and stresses individual consent rights as the way to protect privacy.^66^ This convergence, however, was never complete. Medical privacy law—the law governing privacy in hospitals, clinics, and other clinical health care settings—stubbornly rejects control-over-information theory.^67^ When you enter a clinical health care setting, the legal reality is that you will have very limited control over your information.^68^ Most medical privacy laws, including the Health Insurance Portability and Accountability Act (HIPAA) Privacy Rule,^69^ allow secondary use of data if the patient authorizes (consents to) the use or if the data have been de-identified, but those are just two among many other data-sharing pathways.^70^ The HIPAA Privacy Rule, for example, creates 27 legal pathways for secondary use or sharing of people’s health data, of which 22 require no consent.^71^

This situation is not a uniquely American ‘Wild West’ aberration. The European Union’s General Data Protection Regulation (‘GDPR’),^72^ which many Americans tout as a paragon of strong consent norms, grants the EU Member States leeway to establish their own medical privacy laws.^73^ The Member States’ laws enable many of the same unconsented data flows that the HIPAA Privacy Rule allows.^74^ Data flows, including some unconsented ones, prove unavoidable in order for health care systems to function and serve their essential contextual values—for example, treating the sick, tracking and managing epidemics, detecting domestic violence, facilitating organ transplants, and enforcing health care quality standards.^75^

Medical privacy law ‘regards autonomy as only one of a number of moral principles governing the doctor-patient relationship’ and requires a ‘better balance of patient rights and physician responsibilities’.^76^ Unlike most modern information privacy laws, medical privacy law does not focus on restricting the ‘upstream’ collection of data into the clinical health care system; instead, it follows a ‘downstream data protection model (“confidentiality”)’ that limits disclosure of clinical information *after* the health care system has already collected or generated it.^77^ This orientation reflects the reality that broad collection of patient data is a necessary part of providing health care, and patients will more willingly share needed data with their providers if the latter are bound by confidentiality duties restricting downstream data redisclosure. As already noted, medical privacy laws do allow downstream redisclosure of clinical data for a variety of purposes without consent.^78^ The allowed disclosures are for a narrow list of enumerated purposes that have high social importance and take place in a context where fiduciary duties and professional norms discourage overdisclosure.^79^

The diminished role consent plays in medical privacy law may in part reflect skepticism about whether an ‘upstream’ consent model (seeking consent at the point of data collection) can protect data privacy in clinical settings.^80^ When the PPSC recommended, late in the 1970s, that use of health data should require individual consent, this recommendation was for data used in biomedical research, not for clinical health care.^81^ Free, uncoerced consent can be a meaningful concept in research settings as traditionally conceived, where people weighed whether to contribute their data to a research study that, by definition, had no therapeutic aims.^82^ In contrast, people enter the clinical health care setting ill, in pain, and needing care, sometimes urgently. They seemingly would agree to any data use furthering the health care they are seeking, and their desperation undercuts consent as a tool for protecting data privacy in clinical contexts.

In clinical health care, the longstanding approach for protecting privacy—dating not just to the early 1970s but 2400 years back to Hippocrates—requires health care providers to ‘act as entrusted fiduciaries’ for patients, who are considered too vulnerable to meaningfully self-protect their privacy by refusing to share data with their doctor.^83^ Medical privacy law never embraced the late-20th-century optimism that ‘autonomy as a construct’ can ‘replace the ethical responsibilities of the caregiver’.^84^ Is it better to have people consent to do business with privacy violators, or to impose duties for data handlers not to violate people’s privacy? Medical privacy law took the latter approach, although there are grounds to debate whether the duties it imposes are adequate.^85^ Consent remains hallowed in medical circles as a way to demonstrate respect for persons, but without the illusion that consent is a two-for-the-price-of-one proposition that simultaneously protects their privacy.

The United States relies on a web of state and federal laws defining responsibilities of clinical data handlers. State medical records laws govern the collection, use, and retention of data from medical treatment encounters and set procedures for sharing the records and for their disposal or transfer if the physician/patient relationship ends.^86^ State courts enforce common-law duties for health care providers (eg, physicians, nurses, clinics, hospitals) to protect confidential information they hold, and most states supplement their common law with statutes addressing the privacy of various kinds of data held by health professionals and facilities.^87^ Providers’ duties of confidentiality extend not just to their patients’ data but to information about other people, such as a patient’s family members, that sometimes makes its way into a patient’s files.^88^

General health laws that seem on their face to be unrelated to privacy incorporate significant privacy protections. For example, hospitals breaching patient confidentiality would violate hospital accreditation standards, state facility license terms, and conditions of participation in the Medicare program.^89^ The resulting sanctions could put a loose-lipped hospital out of business. State professional licenses for physicians and nurses focus mainly on professional skills and competence but also address patient confidentiality; violations can lead to loss of licensure.^90^ Nongovernmental bodies such as the American Medical Association add an additional layer of soft law confidentiality norms.^91^ At the federal level, the HIPAA Privacy Rule preempts weaker state medical privacy laws while letting more stringent state provisions continue in effect.^92^ Other federal regulations, commonly called the ‘Part 2 regulations’, protect information generated at or received from federally assisted alcohol and drug abuse treatment programs.^93^

All these laws, together, govern data privacy in one context: clinical health care.^94^ AI/ML CDS tools, designed for that context, are subject to those laws. Data handlers operating AI systems elsewhere—for example, in research settings or in the broader surveillance society—are not bound by the same ethic of responsibility/fiduciary duties of health care providers. Outside clinical health care, control-over-information theory dominates although the ‘control’ it provides is often illusory.^95^ The consents on which it relies are questionable: a recent survey found 97 per cent of Americans recall consenting to a company’s privacy policy but only 9 per cent reported reading the underlying privacy policies to which they agreed.^96^ In 2017, one Wi-Fi company humorously proved ‘the lack of consumer awareness of what they are signing up to’ by inserting a clause in its privacy policy requiring 1000 hours of community service to clean toilets and relieve sewer blockages.^97^ People consented.

Surveys show that people in the United States, Europe, and other technologically advanced societies yearn to control access to their data and believe consent and de-identification of data provide meaningful privacy protection.^98^ Widely reported re-identification attacks over the past 15 years may have shaken their faith that de-identifying data protects privacy.^99^ The response has been to double down on calls for consent: for example, by demanding consent for de-identified as well as identifiable data or passage of GDPR-themed legislation that many Americans hope might strengthen their consent rights. The effectiveness of consent as a privacy protection is rarely questioned despite being questionable.^100^

Data handlers are major beneficiaries of control-over-information theory, which visits the burden of privacy protection not on the data handler but on autonomous individuals, who are expected to research privacy policies, shun risky data handlers, and protect their own privacy as best they can. Control-over-information theory is morally similar to telling the poor to pull themselves up by their bootstraps when, in reality, poverty (and privacy loss) may be systemic to modern information economies.^101^ Prof. Balkin’s first rule shifts the burden of data protection back to data handlers.

The White House Blueprint for an AI Bill of Rights, by dismissing context, overlooks the crucial role fiduciary duties and professional norms play in protecting privacy in the clinical care context. As a result, the Blueprint does not address the salient privacy challenge of AI-enabled health care. Introducing AI/ML CDS tools into the clinical treatment encounter exposes patient data to a new set of actors, such as software vendors and information service providers, who are not bound by the same fiduciary duties and confidentiality norms governing traditional health care providers. Federal action to strengthen patients’ consent rights would do little to protect patients’ privacy, without corresponding reforms to the framework of state laws and soft law norms that require parties who handle clinical data to be careful with it. Grand, sweeping statements about rights, such as those outlined in the Blueprint, belie the grinding work that lies ahead to craft tailored legal protections that can effectively protect rights in the various contexts where AI systems are going to be deployed. Oversimplifying a problem rarely solves it.

### II.C. Rules at the Interface of Robots and Humans

Professors Balkin and Pasquale conceive ‘robots’ as including both mechanical robotic systems and stand-alone AI/ML software, and this article follows their convention.^102^ Robots without intelligence are morally equivalent to mechanical wind-up toys. The concern with robots is not their embodiment, but their ensoulment with something resembling (albeit faintly) human intelligence.^103^ There are contexts where robotic embodiment might be legally significant—for example, if a robot has a fist that punches you in the face, as opposed to running an algorithm that merely advises a human intermediary to do so—but this is not such a context.

By design, AI/ML CDS tools offer recommendations to human health care professionals who can critique, reject, or follow the robot’s recommendations.^104^ Software developers and vendors have little incentive to replace human professionals with robots, when keeping physicians in the loop provides well-insured decoys for tort lawyers to chase, possibly luring them off the scent of software vendors. Moreover, it lightens regulatory scrutiny at a time when, to date, only one fully autonomous AI/ML diagnostic tool has successfully emerged from the Food & Drug Administration’s (FDA’s) premarket review process.^105^

This section judges rules for robots by their capacity to address three challenges a recent report by the U.S. Government Accountability Office and National Academies of Science, Engineering, and Medicine deems critical to the success of AI/ML CDS tools.^106^ The challenges are: ‘protecting privacy’, ‘accessing high-quality data’ to train AI, and reducing ‘potential biases in data’ which can cause CDS tools to give unsafe recommendations for population subgroups underrepresented in the training data. To be fit for purpose in medical AI, any workable set of rules must, at a minimum, address those three challenges.

#### 1. Managing externalities

Prof. Balkin’s second and third rules touch important concerns with AI in the modern surveillance society but seem less well tailored to AI-enabled health care. They address the potential for AI/ML tools to inflict negative externalities (harms) on people who are not involved in developing, using, or having their data processed by the software.^107^ For example, a social media provider that manipulates its end users to swing a national election harms not only its users (who enjoy the benefits of the services provided) but everybody in the nation, including bystanders who gain no benefits and are only harmed.^108^ Prof. Balkin’s second rule places users of AI/ML algorithms under ‘public duties’ to uninvolved parties ‘who are not clients, customers, and end-users’.^109^ The third rule states, ‘The central public duty of algorithm users is to avoid externalizing the costs (harms) of their operations’.^110^

For AI/ML CDS tools, I argue, the central ethical concern is not externalities. Both the benefits and the harms are internalized, and in a way that could exacerbate health care inequities. The harms of AI/ML CDS software fall entirely on *involved* parties (‘clients, customers, and end-users’ of the software). Patients who are not treated using AI/ML CDS tools cannot be injured by their bad advice. Adverse impacts of operating CDS software flow to people connected, one way or another, to its operations. Those impacts include, for example, privacy risks for people whose data are used to train, validate, and operate the software; safety concerns for patients diagnosed and treated using CDS tools; and liability, reputational, and economic risks for healthcare systems and providers when CDS tools contribute to patient injuries. Uninvolved third parties will not experience these harms.

The benefits of AI/ML CDS tools are also internalized. The medical benefits flow to people included in the training data (either directly through inclusion of their own data, or virtually through inclusion of people ‘like’ themselves demographically, medically, socioeconomically, and in terms of race, gender, and environment).^111^ These tools may not be fit for purpose in patients who were not well represented in the training data used when developing the tools.^112^ Stated otherwise, CDS tools exhibit selection bias.^113^

Men of European ancestry were disproportionately included in the training data for many of today’s CDS tools, which tend to perform better for patients who are white males than for women and other racial and ethnic groups treated using the software.^114^ Common data processing practices, such as sorting data into a rigid male/female sex binary, can ‘erase’ patients whose sense of personal identity and gender differ from the sex assigned at birth.^115^ The resulting AI tools can overlook medically significant risks, such as an elevated risk of breast cancer in transgender males or of aortic aneurysm in transgender females.^116^ AI/ML tools developed in high-resource health systems serving privileged, well-insured patient populations can turn unreliable when rolled out to other health care facilities.^117^ Systems trained on data from individuals in high-income countries—that is, virtually all of today’s AI/ML CDS tools—may perform badly for users in low- and middle-income settings.^118^

Rules for managing externalities break down and are not the right rules for AI/ML CDS tools, where the devil lies in the internalities, not the externalities. Even so, Prof. Balkin’s rules offer a useful framing to guide policy. His crucial insight lies in setting distributional justice in the foreground of issues to be addressed by rules for robotic and AI systems. Justice was the neglected third Belmont principle in the ‘lopsided table’ of late 20th-century autonomy-centric bioethics.^119^ For AI/ML CDS tools, the right rules are those that mitigate selection bias, which is shaping up to be the central justice issue in AI-enabled health care.

Selection bias arises in health data sets because people willing to consent to secondary use of their health data differ *medically* from non-consenters—a fact that has been well documented for over 20 years.^120^ Newer evidence suggests consenters also differ *demographically*, which suggests the medical errors would not be randomly distributed, but rather could fall in patterns resembling invidious health care discrimination.^121^ Post-Belmont bioethics, when it engaged with selection bias at all, tended to question whether it materially affects scientific results^122^ (or, when conceding it is material, discounted its ethical importance^123^). These dismissive views no longer work in the age of AI-enabled clinical health care, where biased training data can exacerbate racial, ethnic, gender, and socioeconomic disparities in the quality of care.^124^ If consent norms are feeding selection bias, that is a major ethical concern that can no longer be dismissed.

A complicating factor is that consent norms are just one of many contributors to a much larger problem with biased training data.^125^ Much of the bias observed in today’s CDS tools reflects structural and systemic inequities in the broader economy and health care system.^126^ People who are systematically denied access to health care do not generate clinical health data that can later be harnessed to train CDS tools. Such people are underrepresented in AI training data, not because of the consent norms, but because their data do not exist.

Other aspects selection bias, however, are influenced by the privacy norms a society adopts. For example, rules to anonymize data can strip away important details—such as residential zip codes—that might help detect non-inclusivity and bias.^127^ Notice-and-consent norms can fuel future health inequity if, for example, people of color who have had bad prior experiences with biomedical research selectively decline to participate in AI training data.^128^ Rule-dependent contributions to bias are a worthy focus for empirical analysis, critique, and reform.

Notice-and-consent privacy norms invite the same critique that has been leveled at legal liberalism more generally: they assume ‘all persons share certain “samenesses,” such as rationality and autonomy’ and they allegedly advance a ‘highly specific model of personhood that was constructed for a white male elite’.^129^ When inclusion in training data requires consent, this can have unintended effects that oppress groups for whom the exercise of autonomy (eg, deciding whether share their data to train AI/ML CDS tools) carries group-specific risks (eg, deportation if the data later leak to immigration authorities, or prosecution for gynecological care after *Dobbs v Jackson Women’s Health Organization*).^130^ The Belmont Report and the flowering of bioethical literature in its wake were largely the work of white scholars, and the field allegedly exudes white normativity. ^131^

AI/ML CDS tools challenge core assumptions of 20th century bioethics. For example, bioethics portrays people who contribute data to biomedical research as ‘altruistic’.^132^ Consenters who wrongly believe research will benefit their own health are said to have ‘therapeutic misconception’ and should receive clear notice, before consenting, that the benefits of research, if any, flow to others and not to data contributors.^133^ AI/ML CDS tools upend this altruism narrative: the benefits of participating in training data are internalized, not externalized to others.^134^

The altruism narrative, at heart, rests on an assumption of human sameness: contributing your data to research benefits others (and thus is ‘altruistic’) only if your data are representative of those other people you were hoping to help—that is, if humans share sameness. In clinical care contexts, people were never the same, as Sir William Osler remarked 130 years ago: ‘If it were not for the great variability among individuals, medicine might as well be a science and not an art’.^135^ Modern studies of selection bias prove Osler was right.^136^ In the mid-20th century, when bioethics was born, FDA’s clinical drug trials studied small groups of consenting volunteers, pretending they represented the entire human population and chalking it up to random ‘noise of human variability’ when FDA-approved products tested on largely male, largely white trial populations injured other patients.^137^ The pretense is no longer defensible. The 21st-century health care system is serving an ever-more-diverse patient population and needs CDS tools trained on inclusive data representing all of us. Notice-and-consent privacy schemes, with their sameness presumptions, may be incompatible with that goal.

Prof. Balkin proposes an alternative: a duty-based privacy protection framework in which data handlers act as information fiduciaries. This aligns with the approach medical privacy law has embraced (even if the fiduciary duties it imposes may need strengthening for AI-enabled health care).^138^ For AI software operating elsewhere in the information economy, perhaps control-over-information privacy theory is still appropriate; this article takes no position on that. However, if control-over-information theory truly is the ‘leading paradigm’ that guides the White House Blueprint for an AI Bill of Rights, the case for medical exceptionalism seems strong.^139^

Privacy is only one of five values the Blueprint seeks to advance. The others are safety and effectiveness of AI systems (which requires broad access to high-quality data during system development, validation, and regulatory oversight of real-world system performance);^140^ equity and non-discrimination (which requires inclusive data for ongoing monitoring of system performance and to afford remedies to persons injured by inequitable software);^141^ system transparency;^142^ and human intermediation and oversight.^143^ In domains where selection bias is well-documented,^144^ as in health care, the Blueprint’s notice-and-consent privacy scheme could undermine its other goals of consumer safety, justice, and equity.

The Blueprint resists balancing of public and private interests: system developers and operators should ‘seek your permission’ to use your data, and the social costs of restricting data access in this way are not part of its analysis.^145^ The Blueprint acknowledges that certain contexts may require ‘exceptions’ to ‘balance competing public interests’ but focuses this remark on law enforcement, not health care.^146^ Competing interests are not rare ‘exceptions’ but rather are the rule in any context where the use of personal data confers important benefits on society. Health care is one such context. The American Data Privacy and Protection Act that Congress has been developing recognizes this fact.^147^ As currently drafted, it leaves in place the existing framework of state and federal medical privacy laws as well as various other sectoral privacy schemes addressing concerns in specific data-use contexts.^148^ The Blueprint should embrace this same approach. Its notice-and-consent privacy scheme offers only weak privacy protection^149^ while undermining other important social values that the Blueprint seeks to advance.

#### 2. Etiquette in robot/human relations

Frank Pasquale’s rules address moral and legal concerns about the way robotic and AI/ML systems, especially the more-fully-autonomous ones, interface with human beings. He enunciates four laws of ‘complementarity, authenticity, cooperation, and attribution’ that encourage developers, operators, and users of AI/ML software to ‘develop policies that capitalize on human strengths…and bound the scope and intensity of conflict and regimentation in social life’.^150^ The rules are: (i) ‘Robotic systems and AI should complement professionals, not replace them’;^151^ (ii) ‘Robotic systems and AI should not counterfeit humanity’;^152^ (iii) ‘Robotic systems and AI should not intensify zero-sum arms races’, whether in military, policing, credit-scoring, litigation, social control, or other contexts ‘where people compete for positional advantage’;^153^ and (iv) ‘Robotic systems and AI must always indicate the identity of their creator(s), controller(s), and owner(s)’.^154^

This article discusses AI/ML CDS tools which, by design, assist rather than replace healthcare professionals. This focus sidesteps many of Pasquale’s concerns, so this is not a good forum to do justice to his sparkling insights. Certainly, his concerns would be relevant if a CDS tool *designed* to complement healthcare professionals was *applied* in ways that counterfeit them, for example, by professionals using the tool off-label and passing its recommendations off as their own without informing the patient they are relying on it. State medical practice regulators, the FDA, healthcare accreditation bodies, and medical ethicists should develop policies to limit this and other potential misuses of AI/ML CDS tools.

Pasquale’s third law against using AI/ML to ‘intensify zero-sum arms races’ to enhance ‘positional advantage’ strikes closer to this article’s focus.^155^ A simple Google search on the terms ‘zero-sum game’ and ‘health care’ yields many articles about zero-sum games between health insurers and hospitals, between hospitals seeking to cut costs and patients denied care, and between many other players in a complex, budget-constrained healthcare system. Prof. Cohen *et al.* cautioned, early on in 2014, that tools calibrated to optimize the health of a hospital’s entire patient population or to lower its costs might sacrifice the well-being of individual patients.^156^ Transparency is crucial, so patients have notice whether AI/ML CDS tools are optimizing population health or *their* health.

A fairly diligent search found no sources exploring zero-sum games in the acquisition of data for AI/ML training datasets. This silence, I suggest, is because acquiring training data for AI/ML CDS software is a positive-sum game. The more people whose data are reflected in the training data, the better the AI performs for everyone (assuming, of course, equivalent data quality). Making training data more inclusive and representative of today’s diverse patient population does not take anything away from already-well-represented groups. Thus, adding gender-representative data to a male-dominated AI/ML CDS tool does not erode its performance for men; it just makes it perform better for all who are treated using the software. Making AI/ML training data more inclusive is a way AI/ML CDS software developers can honor Pasquale’s third law against zero-sum arms races. If that entails critiquing and modernizing popular notice-and-consent norms, Pasquale’s third law underscores the ethical duty to do so.

## III. DIFFERENT RULES FOR SIMILAR INFERENCES

The notion that AI/ML medical software warrants special rules presumes medical AI is a clear category, distinct from other types of AI. The line between medical and non-medical software grows blurry in an age when AI tools processing non-medical data can draw inferences about people’s health. ^157^ The Blueprint for an AI Bill of Rights treats ‘health’ data as an undifferentiated category, drawing no distinction between the clinical data licensed physicians develop while treating patients *versus* other health-inflected data trafficked in modern information economies.^158^

This Part questions the Blueprint’s approach and distinguishes two categories of AI that process and produce health-related data. In this discussion, the phrases ‘clinical data’ and ‘clinical inferences’ denote health-related information generated and used to treat patients in traditional clinical care settings. The phrase ‘health inferences outside the clinical context’ (HIOCCs) refers to inferences about people’s health drawn by retailers, credit-scorers, employers, fitness trackers, at-home wellness monitoring devices, services, and other non-medical actors in a surveillance society. In terms of content, the information is at times quite similar.

The distinction between clinical health information and HIOCCs turns on intent. Although health-related, HIOCCs are not intended for use in diagnosing, treating, mitigating, or predicting disease or improving individual health. HIOCCs aid decision-making that affects people’s rights but usually not their health, at least not directly or intentionally. For example, HIOCCs guide decisions on loan eligibility, employment, advertising, and law enforcement, such as deciding to fine rather than imprison an elderly offender to avoid burdening prison health care with high-cost geriatric patients.^159^ Such decisions can indirectly affect health—for example, if a home loan denial prevents someone from moving to a new neighborhood with better facilities for outdoor fitness activities—but the data are not intended to inform clinical health care but to assess credit risk.

Related questions of intent define FDA’s jurisdiction to regulate drugs and devices under the Food, Drug, and Cosmetic Act and limit the scope of federal laboratory oversight under the Clinical Laboratory Improvement Amendments of 1988 (CLIA).^160^ Regulators and courts have successfully administered intent-based jurisdictional rules in those and other contexts, and FDA has even codified its approach for determining whether a product is intended for clinical use.^161^ This prior experience suggests that law is well able to wield the intent-based distinction proposed here.

### III.A. All Data Are Health Data in an AI-Enabled World: The Case for Uniform, Content-Based Consent Norms

AI/ML tools at retail stores can infer—with considerable accuracy—whether a young woman is pregnant based on her pattern of purchasing ‘scent-free soap and extra-big bags of cotton balls, in addition to hand sanitizers and washcloths’.^162^ A 70-year-old woman or a male buying those same products would not trigger an inference of pregnancy, unless the software was trained to expect they might be expecting. In a surveillance society, all data are health data in the sense that they can be processed to draw health-related inferences, but the inferences are only as accurate as the training data that taught the algorithm *what* correlates with *what*.

The inference that Terry is pregnant seemingly deserves the same privacy protection, whether it came from a Target™ store’s AI algorithm, from an at-home pregnancy test, or from a clinical diagnostic test ordered by Terry’s health care provider.^163^ Regardless of the source, the information content is similar and has the same sensitivity around disclosure and the same risks of stigmatization or discrimination, such as causing Terry not to get a job offer.

Almost 30 years ago, the Institute of Medicine (precursor of today’s National Academy of Medicine) called for uniform, content-based protections for health data.^164^ Health data implicate historical and cultural taboos about the body and bodily functions.^165^ They reveal behaviors (eg, substance abuse, sexual activity) and conditions (cancer, mental health, pregnancy) that people might prefer not to disclose.^166^ These attributes fuel a popular expectation that health data should be subject to strong consent norms, wherever in the economy the data are stored.^167^ The need for consent, by this view, reflects inherent data characteristics—for example, the type or substantive content of the data (eg, health-related data), the perceived sensitivity (eg, data on sexually transmitted diseases, as opposed to a patient’s blood type), or the data’s perceived re-identifiability (eg, genetic data).

The existing legal framework for health-related AI lacks uniform, content-based protections both for consumer safety or for privacy and other civil rights. The FDA and CLIA regulations, addressing data quality and consumer safety, only apply to AI intended for clinical use.^168^ The HIPAA Privacy Rule and most state medical privacy laws take a sectoral approach, protecting health information generated or used in clinical health care settings (eg, hospitals, clinics, skilled nursing facilities, health insurers) but not in other contexts (including some contexts that seem health-care-related, such as data held by drug and device manufacturers).^169^ When the HIPAA Privacy Rule does apply, it allows covered entities to share health data for a long list of enumerated purposes without individual consent.^170^

The EU’s GDPR appears—at least to casual observers—to impose consistent consent norms on similar data, regardless of who controls the data.^171^ This perception arises because GDPR identifies ‘special categories’ of information that receive heightened protections.^172^ These categories include data that many people characterize as sensitive: data which, if disclosed, might subject a person to discrimination, stigmatization, embarrassment, stress, or other psycho-social harms. Data ‘concerning health’ are one of GDPR’s special categories.^173^

Rather surprisingly, the HIPAA Privacy Rule is not particularly content-based. It protects individually identifiable ‘health information,’ but this is a legal term of art defined in the HIPAA statute, and it does not map neatly onto GDPR’s concept of data concerning health or onto people’s everyday conception of what is health-related.^174^ HIPAA’s definition of health information is surprisingly content-neutral.^175^ Data become health information, for purposes of HIPAA, if the data are ‘created or received by a health care provider…’ and broadly relate to ‘the past, present, or future physical or mental health or condition of an individual’ *or* to ‘the provision of health care’.^176^ That ‘or’ means that information that is unrelated to ‘past, present, or future physical or mental health’ can still be HIPAA-protected health information simply by being communicated during a medical treatment encounter.

If Sally mentions to her doctor in the course of a clinical encounter that she read *War and Peace* last week, this fact technically becomes health information under HIPAA. Sometimes, that fact truly might be health-related (eg, marking Sally’s progress after recent eye surgery), but often it has no clinical significance. Still, if the information makes its way into Sally’s medical records, HIPAA treats it as health information based on the context: the information was received by Sally’s care provider and was related, even tangentially, to the provision of health care (eg, by enhancing physician/patient rapport and building trust). Context, more than content, is what causes facts to become health information under HIPAA and many other medical privacy laws.

With only one exception, the HIPAA Privacy Rule rejects the notion that some types of health information are sensitive and need heightened privacy protections. That exception is psychotherapy notes, which have special protections with strong consent norms, but this exception is narrow.^177^ Otherwise, the U.S. Department of Health & Human Services’ (HHS) Office for Civil Rights (OCR), which administers HIPAA, has resisted pressure to grant special protections for data that many people ‘characterize as “sensitive,” including genomic, cancer, pregnancy, sexually transmitted disease, and mental health test results’.^178^ HHS maintains that designating ‘“sensitive” and “non-sensitive” categories of health information would be a subjective endeavor and would not necessarily result in policies that are in the patient’s best interest’.^179^

Some might disagree, but HHS’s view of data sensitivity has merit. Something as routine as a patient’s blood type might be far more sensitive than HIV or pregnancy status, if blood type reveals misattributed paternity (where the ostensible father is not really a person’s father, a revelation that can expose pediatric patients to a risk of nonsupport or domestic violence).^180^ Sensitivity is subjective and highly context-dependent, making broad, content-based restrictions a dubious way to protect privacy. GDPR’s top-down sanctification of ‘special categories’ of data discounts the crucial roles subjectivity and context play in defining what is sensitive to each of us.

In the United States, HIOCCs and clinical inferences are subject to different consent norms. The same is true in many other parts of the world if one peruses the local medical privacy laws.^181^Apart from privacy, HIOCCs and clinical inferences are under different data-quality and consumer-safety regulations as well. There is a common-sense argument that uniform rules should apply to all AI systems that use or generate health-related data. The remaining sections offer a contrarian view, arguing against uniform rules.

### III.B. The Weak Link between Consent and Personhood for Clinical Health Information

There are centuries-old norms about individual control over one’s own body. Unconsented touching of the human body has long been viewed as a battery (actionable in tort and under criminal law) as well as an offense to human dignity.^182^ This norm against unconsented touching of the body is not context-dependent: unconsented touching is a battery whether committed in a research setting, during clinical health care, in a retail store, or elsewhere.

This does not imply, however, that similar consent norms should apply to health data. Touching data *about* a person’s body is not the same thing as touching the person’s body. Bioethicists and medical privacy scholars have struggled (not always successfully) to theorize the individual’s right to exert strong control over health data describing oneself. The declared basis for this right is a jumble of assertions about respect for persons, autonomy, human dignity, and selfhood, but it is not quite clear how a consent-based privacy scheme advances those things.

The modern claim that controlling one’s health data is integral to selfhood calls to mind Charles Taylor’s lament about impoverished ontological accounts of the modern Self.^183^ Modern culture, in his view, dethroned concepts of a higher or ‘good’ life, such as the Aristotelian notion that health and the human body are not ends in themselves but instruments for value-laden activities such as contemplation or action as a citizen.^184^ Carl Schneider describes a ‘change in attitudes toward health—the elevation of health to a supreme value…taken as the primary moral value of our civilization’.^185^ This view is epitomized by the fitness buff two researchers quoted as saying, ‘Health is everything for me, it is primordial, and I live for it’.^186^ This account of selfhood portrays the human body and its condition as essential to selfhood. Even if you accept that portrayal—and has not the disability rights movement already debunked it?—this portrayal still does not explain how information *about* the body is essential to the Self.

Casting about for a theoretical framework, it has been tempting for bioethicists and medical privacy scholars to borrow the rich theorizations of why control over personal information matters in other contexts. A particularly enticing body of work explores ‘surveillance in post-industrial, digitally networked societies’ where personal information is routinely collected, stored, and made visible to others and then algorithmically transformed ‘in the active production of categories, narratives, and norms’ that can land us on no-fly lists, earn us a discount, tag us as risky or at-risk human beings, or cause a prospective employer to rule us out.^187^ Some of these works try to explain why flows of personal information and a person’s ability to control them might influence individual self-development.^188^

Often drawing on Michel Foucault’s work on prisons, studies of surveillance portray it as a technique of social discipline, in which access to our data is embedded in the very design of commercial and social institutions^189^ and our data feed statistical analyses that norm us relative to one another as opposed universal norms.^190^ Surveillance ‘operates on its subjects not only by the “normalized soul training” of Foucauldian theory, but also by seduction’ including ‘a cornucopia of benefits and pleasures, including price discounts, social status, and voyeuristic entertainment’ that induce people (willingly or unwittingly) to share data enabling the surveillance.^191^ These works state plausible mechanisms through which a lack of control over one’s data might really affect one’s Self.

Julie Cohen describes an additional strand of surveillance studies based on performance theory, which stresses that human identities are not fixed and invariant across all circumstances, but vary as people play different roles to different audiences.^192^ Stated otherwise, identity is a state rather than an enduring trait.^193^ ‘The struggle for privacy is recast as the individual’s effort to assert multiplicity and resist “norming.”’^194^ The Self to be protected here sounds of Walt Whitman’s internally contradictory, multitudinous Self: ‘Do I contradict myself?/Very well then I contradict myself./(I am large, I contain multitudes.)’.^195^ Under this model of selfhood, it is plausible that the inability to control access to one’s data might damage the Self, by holding people to account for having performed a different Self 15 minutes ago.

Insights from these works enrich but also confuse discussions of medical privacy. Health care providers collect, store, and process personal data not as a means of social discipline or social control, but to try to cure our sick bodies to avert the untimely demise of our selfhood. Clinicians analyze our data and norm us relative to other patients, not as part of ‘normalized soul training’^196^ but to assess, for example, whether we are different enough from other patients who had strokes while taking Rofecoxib (Vioxx™) that the drug might ease our pain without harming us.^197^ Research that uses health data is defined, by federal regulations, as a systematic search for ‘generalizable knowledge’—a quest for universal norms of scientific truth rather than Foucauldian norming as a means of social control.^198^

As for performance theory, multiplicity and duplicity are strongly discouraged as hindering the goals of clinical health care. If a mother with Münchausen Syndrome by Proxy wants to perform an identity in which her child has cancer when the child does not have cancer, we all want the pediatrician to detect and halt that performance of Mom’s selfhood, including by sharing information with other health care providers, with Children’s Protective Services, and with law enforcement.^199^ The arguments that persuade us that consent is essential to selfhood in the broader surveillance society are not well matched to the goals of clinical health care.

### III.C. Medical Privacy Law as a Contextual Privacy Scheme

The crux of a surveillance society, in the popular imagination, is that one’s data are being gathered, analyzed, scored, and potentially critiqued to one’s detriment almost everywhere. The problem is contextual but at a level of generality where the context of surveillance swallows almost every aspect of one’s life except when one stays home hiding under the bed unaccompanied by a geolocation-tracking smartphone.

Medical privacy laws, in contrast, are designed around one specific context, clinical health care.^200^ They implement Helen Nissenbaum’s concept of privacy as ‘contextual integrity.’^201^ As she describes it, contextual integrity is not a definition of privacy or a theory of why privacy matters.^202^ This sidesteps the need to theorize why consent to the release of a person’s health information is essential to selfhood. Every context has distinct ‘informational norms’, a set of expectations about what can appropriately be disclosed, used, or shared with others, and when, to whom, and on what terms.^203^ Contextual integrity—privacy—exists when actual information flows align with those norms.^204^

People and their data do not exist in ‘an undifferentiated social world, but as individuals in certain capacities (roles), in distinctive social contexts, such as health care, education, employment, the marketplace, and so on’.^205^ Information flows appropriate in one context might be inappropriate, and spark protests in the name of privacy, in others.^206^ It is inoffensive for a sales associate in the lingerie department to inquire about your bra size; the same question would be off-putting when applying for a home loan. A content-based conception of data privacy (for example, the idea that some data are inherently ‘sensitive,’ requiring uniform protection in all circumstances) fails, because the same piece of information might be permissible to share in one context but not in another.

This resonates with Prof. Balkin’s point about shifting the focus of privacy discourse ‘from the kind of *information* to the kinds of *relationships* that produce the information’ and constrain information flows within and beyond those relationships.^207^ Production of health data in a physician’s office takes place in a relationship of trust with (fairly) clear norms limiting what can be done with the data.^208^ In contrast, production of health data by a retailer processing customers’ purchase data takes place in a different relationship with fewer (if any) constraints on how the data might be used.

The social relationships that Prof. Balkin emphasizes are only one part of Nissenbaum’s contexts. A context also includes a set of ends and values that people serve by entering that context and forming those relationships.^209^ Clinical health care, for example, serves the ends of diagnosing and treating illnesses and protecting the public’s health—ends that most people consider important.^210^ These ends and values (and not just the relationships people form to pursue them) affect privacy policy.^211^

By admitting contextual ends and values exist, Nissenbaum adds an element often missing in discussions of privacy policy. As Carter Snead notes, ‘American public bioethics is strongly anti-teleological. It does not recognize natural “ends” that guide understanding of the flourishing of the individual human’ and focuses instead on ‘the self-defining projects of the individual will’.^212^ Yet, in reality, contexts are teleological: they serve purposes.

The ends and values of a context influence its informational norms by informing the trade-offs that occasionally must be made, in any context, between the moral virtues of keeping data secret versus the moral virtues of data disclosure. Information disclosures that might be ethically justified to serve important ends and values in one context, such as clinical health care, might be inappropriate in other contexts where disclosures serve other and potentially less noble ends, such as serving unwanted advertisements to Internet users, denying someone a home loan, consigning people to no-fly lists, or targeting people for deportation. Each context has its own ends, and the ends justify—or fail to justify—a particular data disclosure.

This reasoning leads to a counterintuitive result: strong notice-and-consent privacy norms may be *more* justified in contexts that serve *less* important ends because, in such contexts, the moral value of unconsented disclosures is lower and less able to outweigh the individual’s interest in non-disclosure. By this view, the ethical justification for notice-and-consent privacy norms is *inversely* proportional to the importance of the ends and values a context serves. In clinical health care, unconsented access to my health data might be well justified, for example, if it helps doctors save another patient who is in immediate danger, or helps them track an epidemic that threatens public health. There is less (but still some) justification to use my health data without consent in biomedical research: research serves a valuable end—helping other patients—but does so with uncertainty and usually after years of delay. Continuing down the hierarchy of ends, unconsented data uses are least justified in the surveillance society *writ large* where others seek to use my health data for ends that are sometimes frivolous, unimportant, or downright harmful.

By this reckoning, notice-and-consent norms make more sense in the information society generally than in the special context of clinical health care. This utilitarian balancing goes against the weight of post-Belmont bioethical literature in which individual autonomy trumps competing moral principles.^213^ Nissenbaum’s work is not a normative defense of balancing, but a pragmatic recognition that in every context, specific ends are being served and moral trade-offs do take place. Clinical health care is a context where, in practice, the moral value of data disclosures (for justice, beneficence, and non-maleficence) carries considerable weight.

### III.D. The Role of Context in Establishing Rules for AI Tools

If medical and non-medical AI warrant different rules, this does not mean that HIOCCs deserve less protection than clinical inferences receive.^214^ Three factors, discussed earlier, suggest that HIOCCs may deserve *stronger* consent requirements than law requires for clinical health information. Those factors were: (i) differences in the underlying social relationships and weaker fiduciary duties of data handlers outside the clinical context, (ii) differences in the values data disclosures serve, and (iii) concerns that consent norms can contribute to selection bias, and the gravity of those concerns in clinical contexts where biases can feed health care inequities.^215^

A fourth factor is data quality. Medical privacy law embraces disclosure-friendly informational norms allowing unconsented disclosures of clinical information for a long list of purposes.^216^ Similar informational norms might be highly inappropriate, allowing too much disclosure of health-related data, elsewhere in the surveillance society. Information generated in clinical care settings is subject to regulatory and professional standards aimed at ensuring data quality and accuracy. Clinical records sometimes do contain errors (even serious ones), but law creates strong incentives favoring accuracy. In contrast, HIOCCs derived from a person’s purchase data, Internet browsing history, or geolocation generally do not meet clinical standards of data quality. HIOCCs can be misleading and even (wildly) inaccurate. If stigmatization and discrimination based on people’s well-validated clinical health information is repugnant, then it is even more concerning to curtail people’s rights based on the dubious, low-quality surmises of poorly validated non-medical AI tools. Granting people a consent right alerts them that their HIOCCs are being circulated, possibly giving them a chance to dispute the information if it is inaccurate.

A fifth factor concerns ownership and control of inferences derived from health data. The HIPAA Privacy Rule and state medical privacy laws grant individuals a right of access not only to their raw personal data (eg, medical images and test results) but also medical opinions and inferences drawn from the data (eg, this person has cancer and is most likely to respond to Drug X).^217^ HIPAA-covered entities *must* provide such access if patients request it.^218^ Elsewhere, it can be hard for people to gain access to inferences about themselves, even when laws such as the EU’s GDPR provide access rights to data.^219^ Under GDPR, people’s raw personal input data might be accessible, but the personal inferences often are not.^220^ Such policies recognize the legitimacy of competing claims to inferential knowledge. Inferences are not fully derivative of the input data on which they are based but also incorporate investments of effort, skill, and expertise. Under a Lockean ‘desert’ theory of ownership, people who draw inferences might have as legitimate a claim to an inference as does the person the inference describes.^221^ Medical privacy law treats clinicians’ inferences as a sort of work-for-hire that the patient is entitled to know; this flows from healthcare providers’ fiduciary ‘duty of care’ to act in patients’ best interests, and from the fact that clinical inferences potentially affect patients’ health, which is the contextual value served by medical privacy laws.^222^

Similar handling of inferences would not necessarily be appropriate in general data processing contexts, where people’s raw personal data are processed to extract valuable insights and discoveries that might or might not have direct utility to the individual. As Lawrence Lessig points out, ‘No one spends money collecting these data to actually learn anything about you. They want to learn about people like you’.^223^ When that is true, data processors might have legitimate claims to their insights and discoveries, with individual having lesser interests in the inferences drawn from their data.

A final factor concerns concordance with traditional ways of making decisions about matters of public safety. Clinical health care obviously affects public safety, and the HIPAA Privacy Rule’s norms on unconsented data flows were designed to enable a set of ‘national priority’ activities that HHS determined were matters of public concern.^224^ The HIPAA Privacy Rule is a notice-and-consent privacy regulation only in a Blackstonian sense of consent: when a duly elected Congress empowers a federal regulator to decide which data uses are in the public’s interest, there is Blackstonian ‘consent of the people’—a consent produced by collective rather than individual decision-making.^225^

Collective consent is often used in deciding matters of public safety and concern. For example, modern societies routinely confide decisions about traffic safety to legislative decision-making, and once made, such decisions are binding on every member of the driving public with or without individual consent to observe the speed limit and obey traffic signals. Philosopher Charles Taylor acknowledges that one could argue that it violates individual autonomy to be forced to stop at traffic light, but ‘not in a serious political debate’.^226^ ‘[I]in such a case it is incorrect to speak of an infringement of freedom: the security and convenience of the walkers are in question, not freedom.’^227^ People perceive infringements on freedom ‘against a background of understanding that certain goals and activities are more significant than others’.^228^ This accords with Nissenbaum’s insight that contextual ends and values are crucial points to consider when setting privacy policy.^229^ In contrast, flows of HIOCCs in the broader surveillance society often are not matters of public safety or concern. While it goes against our intuition, this fact strengthens the case for individual control over HIOCCs.

## IV. CONCLUSION

This article chose AI/ML CDS tools as a metaphor for many other AI tools that will raise context-dependent ethical and legal issues in the future. It identified contextual factors that justify exceptionalism in clinical data privacy. Despite continuing calls to embrace control-over-information theory, medical privacy law stubbornly clings to fiduciary duties of data handlers, rather than consent, as its main tool of privacy protection. While the overall approach seems sound, AI-enabled health care strains it by introducing new data handlers (such as commercial software developers) not bound by the framework of laws imposing fiduciary duties on traditional health care providers. Gap-filling reforms are needed to strengthen responsible data handling in AI systems intended for clinical use. ^230^

There is a strong case against extending medical privacy laws to non-medical AI systems that process or produce health-related data. Medical privacy laws allow and, in the case of individual access to data and inferences about oneself require*,* various disclosures. Those same norms might burden people’s privacy unjustifiably, or deny data processors the fruits of their own inferences, in other data processing contexts where the ends, values, data quality, and social relationships are different.

Beyond privacy, rules for robotic and AI systems must also promote justice, recognizing that the salient questions of justice are often context-dependent. The expanding role of AI in society requires us to right the lopsided bioethical table of the past 40 years that stressed autonomy to the near-exclusion of justice and equity. We are no longer in a strictly human-versus-human contest where the person exercising the most autonomy wins. A new contestant has entered the game—AI—and human-versus-AI conflicts will emerge. Humans must practice sticking together and working as a team in the waning hours before AI grows smarter than we are. Sticking together implies cultivating the neglected Belmont principles of beneficence and justice, while there is still time.

## Footnotes

1 The White House, Blueprint for an AI Bill of Rights: Making Automated Systems Work for the American People (The White House Office of Science and Technology Policy, October 2022), https://www.whitehouse.gov/ostp/ai-bill-of-rights/ (Accessed Jan. 22, 2023) [hereinafter, ‘Blueprint’].

2
Isaac Asimov, *Runaround,* in I, Robot 37 (1950) (republishing a story first appearing in the Mar. 1942 issue of the periodical Astounding Science Fiction).

3 See generally Frank Pasquale, New Laws of Robotics: Defending Human Expertise in the Age of AI (2020) (discussing various applications of AI); Christopher Slobogin, Just Algorithms: Using Science to Reduce Incarceration and Inform a Jurisprudence of Risk (2021) (arguing that properly regulated risk-assessment tools can advance reform in the criminal justice system).

4 Carlos Ignacio Gutierrez & Gary E. Marchant, A Global Perspective on Soft Law Programs for the Governance of Artificial Intelligence (May 28, 2021), available at https://ssrn.com/abstract=3855171 (Accessed Jan. 22, 2023).

5 See generally Helen Nissenbaum, Privacy in Context: Technology, Policy, and the Integrity of Social Life (2010); Adam Barth, Anupam Datta, John C. Mitchell & Helen Nissenbaum, *Privacy and Contextual Integrity: Framework and Applications*, 2006 Proc. of the IEEE Symposium on Security and Privacy 184–98 (2006), available at https://papers.ssrn.com/sol3/papers.cfm?abstract_id=2567438 (Accessed Jan. 22, 2023); Helen Nissenbaum, *Privacy as Conceptual Integrity*, 79 Wash. L. Rev. 119 (2004).

6 Bryce Goodman & Seth Flaxman, *European Union Regulations on Algorithmic Decision-Making and a ‘Right To Explanation’,*arXiv (June 28, 2016), https://arxiv.org/abs/1606.08813 (quoting J. Burrell, *How the machine ‘thinks’: Understanding opacity in machine-learning algorithms*, 3 Big Data and Society (2016)) (Accessed Jan. 22, 2023).

7 Office of the National Coordinator for Health Information Technology (ONC), *Clinical Decision Support* (Apr. 10, 2018), at https://www.healthit.gov/topic/safety/clinical-decision-support (describing a range of CDS tools providing decisional support to health care professionals) (Accessed Jan. 22, 2023).

8 Julia Adler-Milstein et al., *Meeting the Moment: Addressing Barriers and Facilitating Clinical Adoption of Artificial Intelligence in Medical Diagnosis* 15 (National Academy of Medicine Discussion Paper, Sept. 29, 2022), at https://nam.edu/meeting-the-moment-addressing-barriers-and-facilitating-clinical-adoption-of-artificial-intelligence-in-medical-diagnosis/ (Accessed Jan. 22, 2023).

9
*Id*.

10 See ONC, *supra* note 7.

11 See U.S. Food & Drug Admin., Clinical Decision Support Software: Guidance for Industry and Food and Drug Administration Staff 10 (Sept. 28, 2022) at https://www.fda.gov/regulatory-information/search-fda-guidance-documents/clinical-decision-support-software (Accessed Jan. 22, 2023) (noting that the Food and Drug Administration (FDA), consistent with language in the 21st Century Cures Act at 21 U.S.C. § 360j(o)(1)(E)(ii), conceives CDS software as tools ‘intended to support or provide recommendations to an HCP [health care professional] about prevention, diagnosis, or treatment of a disease or condition’). *But see* Section 3060(a) of the 21st Century Cures Act, 21 U.S.C. § 360j(o)(1)(B) (leaving the FDA with authority to regulate patient-facing software related to ‘the diagnosis, cure, mitigation, prevention, or treatment of a disease or condition’ although the agency has generally referred to such tools as ‘patient decision support’ rather than CDS tools). See, eg, U.S. Food & Drug Admin., *Clinical and Patient Decision Support Software: Draft Guidance for Industry and Food and Drug Administration Staff* (Dec. 2017) (subsequently replaced by the final guidance published on Sept. 28, 2022, which focuses on CDS software while deleting discussion of patient decision support software).

12 See Bradley Merrill Thompson, *Learning from Experience: FDA’s Treatment of Machine Learning*, Mobile Health News (Aug. 23, 2017), http://www.mobihealthnews.com/content/learning-experience-fda%E2%80%99s-treatment-machine-learning [https://perma.cc/Q95C-9R22] (discussing AI in current diagnostic imaging devices) (Accessed Jan. 22, 2023). Software of this type, in many instances, technically fits within the concept of CDS software that aids a health care professional’s diagnostic decisions, but it is subject to a ‘saving clause’ in the 21st Century Cures Act that unequivocally preserves the FDA’s traditional authority to regulate software ‘intended to acquire, process, or analyze a medical image’ from a hardware medical device. See 21 U.S.C. § 360j(o)(1)(E) and see Alder-Milstein et al., *supra* note 8, at 16. Discourse about such software thus tends to focus on the FDA’s power to regulate it in the manner of traditional medical device software rather than as clinical decision support software.

13 See Adler-Milstein et al*., supra* note 8*,* at 15.

14 The use of a possessive pronoun in the phrase ‘their data’ in this article is relational (ie, data relating to them) and is not intended to imply legal ownership of the data, in the same way that phrases like ‘his country’ or ‘her son’ indicate relationships other than ownership.

15 Rebecca Robbins & Erin Brodwin, *An Invisible Hand: Patients Aren’t Being Told About the AI Systems Advising their Care*, Stat News (July 15, 2020), https://www.statnews.com/2020/07/15/artificial-intelligence-patient-consent-hospitals/ (Accessed Jan. 23, 2023).

16 See, eg, David Lyon, Surveillance Society: Monitoring Everyday Life, 33–35, 114–18 (2001) (discussing pervasive data collection in modern surveillance societies); Frank Pasquale, The Black Box Society (2015) (same).

17
Secretary’s Advisory Comm. on Genetics, Health, & Soc’y [SACGHS], U.S. Dep’t of Health & Human Servs., U.S. System of Oversight of Genetic Testing 17 (2008).

18
*Id*. (discussing the work of the Task Force on Genetic Information and Insurance, formed in 1991 as part of a Joint National Institutes of Health-Department of Energy (NIH-DOE) Working Group on the Ethical, Legal, and Social Implications of Human Genome Research).

19 See Eric Topol, The Creative Destruction of Medicine 114 (2012) (recounting this history).

20 SACGHS*, supra* note 17, at 17–20 (citing and reviewing some of this literature).

21 Charles Duhigg, *How Companies Learn Your Secrets*, The New York Times Magazine, Feb. 16, 2012, https://www.nytimes.com/2012/02/19/magazine/shopping-habits.html (Accessed Jan. 22, 2023).

22 Hanoch Dagan, *Remedies, Rights, and Properties*, 4 J. Tort L. art.3, n. 73 (2011) (referring to the category of property).

23
Nat’l Comm’n for the Protection of Human Subjects of Biomedical and Behavioral Research, U.S. Dep’t of Health, Educ. & Welfare, The Belmont Report: Ethical Principles and Guidelines for the Protection of Human Subjects of Research pt. B (1979), https://www.hhs.gov/ohrp/regulations-and-policy/belmont-report [https://perma.cc/99VW-5ZN6] [hereinafter, ‘Belmont Report’] (Accessed Jan. 24, 2023).

24 See Asimov, *supra* note 2, at 37; Pasquale, *supra* note 3, at 3, 7, 9–10, 11; Jack M. Balkin, *The Three Laws of Robotics in the Age of Big Data*, 78 Ohio State L. J. 1217, 1227 (2017).

25
Asimov, *supra* note 2, at 37.

26 See, eg, SJL Edwards et al., *Ethical Issues in the Design and Conduct of Randomised Controlled Trials*, 2 Health Technical Assessment ii (Dec. 1998) (defining ‘liberalism’ in research bioethics as a ‘set of ideas in social and political thought which underlines the importance of individual rights.’).

27 See Belmont Report, *supra* note 23.

28
*Id.* See also Ruth R. Faden & Thomas L. Beauchamp, A History and Theory of Informed Consent 10 (1986) (explaining that beneficence includes not inflicting harm as well as doing good).

29 Alfred I. Tauber, *Sick Autonomy*, 46 Perspectives in Biology and Medicine 484, 488 (2003). See also Thomas L. Beauchamp & James F. Childress, Principles of Bioethics 5th ed. (2001) (acknowledging that autonomy enjoys a central position that marginalizes beneficence, nonmalfeasance, and justice); Paul Root-Wolpe, *The Triumph of Autonomy in American Bioethics: A Sociological View* 39–59 in Bioethics and Society: Constructing the Ethical Enterprise (R. DeVries & J. Subedi, eds., 1988) (remarking that autonomy ‘indisputably [became] the central and most powerful principle in ethical decisionmaking in American medicine’)).

30 See Tauber, *supra* note 29, at 489.

31 Stanley L. Benn, *Privacy, Freedom, and Respect for Persons*, in Nomos XIII: Privacy 2, 26 (J. Ronald Pennock & J. W. Chapman eds., 1971). See also Daniel J. Solove, *Conceptualizing Privacy*, 90 Cal. L. Rev. 1087, 1116 (2002) (discussing Benn’s view that privacy amounts to respect for individuals as choosers).

32 O. Carter Snead, What It Means to be Human: The Case for the Body in Public Bioethics 71 (2020).

33
Alfred I. Tauber, Patient Autonomy and the Ethics of Responsibility 16 (2005).

34 See Tauber, *supra* note 29, at 489.

35 See *id*. at 484, 488 (discussing how autonomy ‘trumps other contenders’ such a beneficence).

36 See Tauber, *supra* note 33, at 18.

37
*Id.* at 16.

38 Ferdinand Schoeman, *Privacy: Philosophical Dimensions of the Literature*, in Philosophical Dimensions of Privacy 1, 3 (Ferdinand David Schoeman ed., 1984). See also Deborah C. Peel, *Written Testimony Before the HIT Policy Committee*, Electronic Privacy Info. Ctr. (Sept. 18, 2009), https://perma.cc/Q34X-GQZR (framing privacy as ‘control of personal information’) (Accessed Jan. 24, 2022); Paul M. Schwartz, *Internet Privacy and the State*, 32 Conn. L. Rev. 815, 820 (2000) (noting control, rather than secrecy, is central to the modern view of data privacy).

39 See Solove, *supra* note 31, *at* 1111.

40 See Kayte Spector-Bagdady, *Governing Secondary Research Use of Health Data and Specimens: The Inequitable Distribution of Regulatory Burden Between Federally Funded and Industry Research*, J. L. & Biosciences 1, 4 (2021) (discussing the shift from human-subjects clinical research that studies people’s bodies to informational research ‘research with all the stuff [such as data and biospecimens] derived from them.’).

41 Privacy Act of 1974, 5 U.S.C. § 552(a)(d). See Privacy Protection Study Commission, Personal Privacy in an Information Society 280 (1977), available at https://archive.epic.org/privacy/ppsc1977report/ [hereinafter, ‘PPSC Report’] (‘Federal rules governing the funding of medical research require the informed consent of individuals who participate in it as research subjects, but do not require their consent when medical records are reviewed and abstracted for retrospective epidemiological research studies.’) (Accessed Jan. 24, 2022).

42 43 Fed. Reg. 56,174, 56,188 (Nov. 30, 1978).

43 See PPSC Report, *supra* note 41, at 574.

44 Federal Policy for the Protection of Human Subjects of Biomedical Research (‘Common Rule’), 45 C.F.R. §§ 46.101–124 (requiring consent for research uses of data).

45 See, eg, Neil Richards, *The Information Privacy Law Project*, 94 Geo. L.J. 1087 (2006) (describing the Information Privacy Law Project as ‘a group of scholars focused on the legal issues raised by the increasing collection, use, and disclosure of personal information made possible by evolving digital technologies’).

46 See Schwartz, *supra* note 38, at 820.

47 Anita L. Allen, *Privacy-as-Data Control: Conceptual, Practical, and Moral Limits of the Paradigm*, 32 Conn. L. Rev., 861, 867 (2000).

48 Gergely Biczók & Pern Hui Chia, Interdependent Privacy: Let Me Share Your Data 1 (2013), http://fc13.ifca.ai/proc/10-1.pdf [https://perma.cc/WWP2-9VC8] (Accessed Jan. 22, 2023).

49 Marwan K. Tayeh et al., *The Designated Record Set for Clinical Genetic and Genomic Testing: A Points to Consider Statement of the American College of Medical Genetics and Genomics (ACMG)*, Genetics in Medicine, available online ahead of print: https://www.gimjournal.org/article/S1098-3600(22)01026-7/fulltext, doi: https://doi.org/10.1016/j.gim.2022.11.010 (Accessed Jan. 22, 2023).

50 Krystal S. Tsosie, Joseph M. Yracheta & Donna Dickenson, *Overvaluing Individual Consent Ignores Risks to Tribal Participants*, 20 Nat. Rev. Genet. 497–498 (2019) doi: https://doi.org/10.1038/s41576-019-0161-z.

51 Cynthia Dwork et al*., Calibrating Noise to Sensitivity in Private Data Analysis*, 3 Theory Cryptograph. Conference 265 (2006).

52 See, eg, Paul Ohm, *Broken Promises of Privacy: Responding to the Surprising Failure of Anonymization*, 57 UCLA L. Rev. 1701 (2010) (discussing re-identification risk).

53 See Blueprint, *supra* note 1, at 5, 26–27.

54 Franklin G. Miller, *Research on Medical Records Without Informed Consent*, 36 J.L. Med. & Ethics 560, 560 (2008) (describing but not necessarily endorsing this view).

55 See Balkin, *supra* note 24, at 1221; Pasquale, *supra* note 3, at 3, 7, 9–10, 11.

56 See discussion *infra* Part II.C (discussing rules addressing the interface between humans and robots, as opposed to rules addressing privacy).

57 See Balkin, *supra* note 24, at 1227.

58
*Id.*.

59
*Id*.

60
*Id*.

61 Jack M. Balkin, *Information Fiduciaries and the First Amendment*, 49 U.C. Davis L. Rev. 1183, 1187 (2016).

62
*Id.* at 1205.

63 Balkin, *supra* note 24, at 1227.

64 See *supra* notes 21–27 and accompanying text.

65
Tauber, *supra* note 33 at 18 (internal citations omitted).

66 Schwartz, *supra* note 38, at 820. See Solove, *supra* note 31, at 1111.

67 This article uses the customary term ‘medical privacy law’ while agreeing with Prof. Nicolas Terry that the body of laws known by that name are better described as a downstream confidentiality scheme for handling and disclosing data *after* the health care system has already collected or generated the data. See Nicolas P. Terry, *Regulatory Disruption and Arbitrage in Health-Care Data Protection*, 17 Yale J. Health Pol’y, L. & Ethics 143, 151–54 (2017).

68 See, eg, Letter from William W. Stead, Chair, Nat’l Comm. Vital & Health Statistics, to Honorable Sylvia M. Burwell, Secretary, U.S. Dep’t of Health & Human Servs. app.A at 15–19 (Nov. 9, 2016), https://www.ncvhs.hhs.gov/wp-content/uploads/2013/12/2016-Ltr-Privacy-Minimum-Necessary-formatted-on-ltrhead-Nov-9-FINAL-w-sig.pdf [https://perma.cc/ J7DF-X9VP] (listing Privacy Rule provisions allowing unconsented disclosure and use of data) (Accessed Jan. 22, 2023).

69 Health Insurance Portability and Accountability Act of 1996, Pub. L. No. 104–191, 110 Stat. 1936 (codified as amended in scattered sections of 18, 26, 29 and 42 U.S.C.); 45 C.F.R. pts. 160, 164 (HIPAA Privacy Rule).

70 See 45 C.F.R.§ 164.502(a)(1)(iv) (allowing PHI to be released with individual authorization); *id.* § 164.508 (describing requirements for a valid individual authorization, which is HIPAA’s term for a consent). See *id* § 164.502(d) (allowing de-identified data to be used and disclosed without individual authorization).

71 See Barbara J. Evans, *The HIPAA Privacy Rule at Age 25: Privacy for Equitable AI*, 50 FSU L. Rev. (2023 *forthcoming*) available at https://ssrn.com/abstract=4316211 (Accessed Jan. 22, 2023).

72 European Union General Data Protection Regulation (Regulation (EU) 2016/679) [hereinafter, ‘GDPR’].

73 See *id.* Art. 6 (requiring consent for processing of personal data *id. § 1(a)* but allowing unconsented processing for various purposes such as legal compliance, ‘to protect the vital interests of the data subject or another natural person,’ for tasks ‘carried out in the public interest’ see *id. §§ 1(b)–(f)* and allowing Member States to specify provisions ‘to adapt the applications of the rules’ in some of these circumstances). See id. Art. 9 (addressing the processing of ‘special categories of personal data,’ which include health data and requiring consent *id. § 2(a)* but allowing Member States to establish different conditions and safeguards for data used in ‘preventive or occupational medicine, for the assessment of the working capacity of the employee, medical diagnosis, the provision of health or social care or treatment or the management of health or social care systems and services’ *id. § 2(h)* and for public health *id. §2(i)* and for public interest purposes including scientific research *id. §2(j). See also id.* Art. 89 (allowing Member State law to derogate from various rights provided by GDPR when those ‘rights are likely to render impossible or seriously impair the achievement’ of various public-interest goals including scientific research).

74 See European Commission, Director General of Health and Food Safety, Assessment of the EU Member States’ rules on health data in the light of GDPR (Specific Contract No. SC 2019 70 02 in the context of the Single Framework Contract Chafea/2018/Health/03) (European Union, 2021).

75 U.S. Dep’t of Health & Human Servs., *Confidentiality of Individually Identifiable Health Information: Recommendations of the Secretary of Health and Human Services, Pursuant to Section 264 of the Health Insurance Portability and Accountability Act of 1996*, § I.I (Sept. 11, 1997), https://aspe.hhs.gov/report/confidentiality-individually-identifiable-health-information [https://perma.cc/M9TK-YZQW] [hereinafter, ‘HHS, 1997 Recommendations’] (identifying ‘national priority’ data uses, deeming them to be ethically justified without consent, and noting that the U.S. Department of Health and Human Services (HHS) was ‘aware of the concerns of privacy and consumer advocates’ about controlling access to their data, but determined that ‘[t]he allowable disclosures and corresponding restrictions we recommend reflect a balancing of privacy and other social values’) (Accessed Jan. 22, 2023).

76
*Id.* See also Tauber, *supra* note 33, at 18.

77 See Terry, *supra* note 67, at 143.

78 See discussion *supra* notes 69–75 and accompanying text.

79 See discussion *infra* notes 86–94 and accompanying text.

80 See discussion *supra* notes 47–52 and accompanying text.

81 See PPSC Report*, supra* note 41, at 574.

82 See 45 C.F.R. § 46.102(l) (defining research as a ‘systematic investigation…designed to develop or contribute to generalizable knowledge’ as opposed to treating a sick patient). See also 45 C.F.R. § 164.501 (mirroring this same definition in the HIPAA Privacy Rule). See also Lynn A. Jensen, The Ethics of Altruism in Clinical Research, 39 Hastings Center Report 26, 26–28 (2009), available at https://www.jstor.org/stable/25548408?seq=1&cid=pdf-reference#references_tab-contents (expressing the traditional view that research participation ‘offer[s] little direct therapeutic benefit and expose[s participants] to significant risks of harm’) (Accessed Jan. 22, 2023). But see Nancy E. Kass et al*., The Research-Treatment Distinction: A Problematic Approach for Determining Which Activities Should Have Ethical Oversight*, 43(S1) Hastings Center Report S4*–*S15 (2013) (questioning the ongoing validity of the research/clinical care distinction in the age of personalized medicine and a learning health care system) and see Ruth R. Faden et al*., An Ethics Framework for a Learning Health Care System: A Departure from Traditional Research Ethics and Clinical Ethics*, 43(S1) Hastings Center Report S16-S27 (elaborating ethical principles for this altered environment).

83 See John C. Moskop et al., *From Hippocrates to HIPAA: Privacy and Confidentiality in Emergency Medicine—Part I: Conceptual, Moral, and Legal Foundations*, 45 Ann. Emerg. Med. 53, 53 (2005) available at https://www.ncbi.nlm.nih.gov/pmc/articles/PMC7132445/ (ascribing to Hippocrates the oath, ‘What I may see or hear in the course of the treatment or even outside of the treatment in regard to the life of men, which on no account one must spread abroad, I will keep to myself, holding such things shameful to be spoken about.’) (Accessed Jan. 22, 2023). *See*Tauber, *supra* note 33, at 18 (describing the traditional medical ‘ethic of beneficence’ in which physicians ‘act as entrusted fiduciaries’).

84 See Tauber*, supra* note 33, at 18.

85 See, eg, Evans, *supra* note 71 (critiquing the HIPAA Privacy Rule’s norms for unconsented data disclosures and proposing reforms to strengthen the fiduciary duties imposed on entities such as software developers and vendors that routinely will be handling clinical data in an AI-enabled health care system).

86 See generally P. Jon White & Jodi Daniel, *Privacy and Security Solutions for Interoperable Health Information Exchange Report on State Medical Record Access Laws* (August, 2009), at https://www.healthit.gov/sites/default/files/290-05-0015-state-law-access-report-1.pdf (providing a multistate survey of state medical records laws) (Accessed Jan. 22, 2023). See, eg, *id*. app.7, https://www.healthit.gov/sites/default/files/appa7-1.pdf (summarizing record retention requirements) (Accessed Jan. 22, 2023).

87
Barry R. Furrow et al., Health Law 161 (8th ed., 2018).

88 See, eg, 45 C.F.R. § 164.524(a)(3)(ii) (calling for redaction of data in a patient’s PHI that ‘makes reference to another person’).

89 See American Medical Association, *HIPAA Violations & Enforcement*, https://www.ama-assn.org/practice-management/hipaa/hipaa-violations-enforcement (noting that a provider’s breach of patient privacy can result in exclusion from the Medicare program, among other sanctions) (Accessed Jan. 22, 2023); The Joint Commission, https://www.jointcommission.org/standards/standard-faqs/hospital-and-hospital-clinics/information-management-im/000001462/ (noting, as part of the standards for accreditation by The Joint Commission, an influential private accreditation body of private health care providers, the need for strict privacy and data security compliance) (Accessed Jan. 22, 2023).

90 See Furrow et al., *supra* note 87, at 117 (enumerating various fiduciary duties of licensed healthcare professionals, including ‘a duty to hold in confidence information learned about a patient in a treatment relationship’).

91 See, eg, American Medical Association, Confidentiality: Code of Medical Ethics Opinion 3.2.1 (undated) at https://www.ama-assn.org/delivering-care/ethics/confidentiality (‘Physicians in turn have an ethical obligation to preserve the confidentiality of information gathered in association with the care of the patient.’) (Accessed Jan. 22, 2023).

92 45 C.F.R. §§ 160.202–.203 (preemption provisions).

93 See 42 C.F.R. §§ 2.1–2.67. See also Kristen Rosati, An Analysis of Legal Issues Related to Structuring FDA Sentinel Initiative Activities 23–24 (eHealth Initiative Foundation, 2009) (providing a useful short summary of the Part 2 regulations).

94 Note that data from HIPAA-covered clinical encounters that makes its way into public-sector databases (eg, Medicare claims data) thereafter is protected not by HIPAA, but by a different statute, the Privacy Act of 1974, which is not specific to clinical data and governs many types of personal information (medical and non-medical) held in governmental databases. See 5 U.S.C.§ 552a.

95 See Schwarz, *supra* note 30, at 820 (calling control-over-information theory the leading paradigm). *Cf.* Evans, *supra* note 71 (reviewing computer science literature on the ineffectiveness of consent as a privacy protection). See Ellen W. Clayton et al*., The Law of Genetic Privacy: Applications, Implications, and Limitations*, J. L. & Biosciences 1, 36 (2019) (calling genetic privacy protections ‘largely a mirage.’).

96
Brooke Auxier et al., Americans’ Attitudes and Experiences with Privacy Policies and Laws (Pew Research Center, Nov. 15, 2019) https://www.pewresearch.org/internet/2019/11/15/americans-attitudes-and-experiences-with-privacy-policies-and-laws/ (Accessed Jan. 22, 2023).

97 Matthew S. Schwartz, *When Not Reading the Fine Print Can Cost Your Soul*, NPR (Mar. 8, 2019), https://www.npr.org/2019/03/08/701417140/when-not-reading-the-fine-print-can-cost-your-soul (Accessed Jan. 22, 2023).

98 See, eg, Comm. on Health Research and the Privacy of Health Info.: The HIPAA Privacy Rule, Inst. of Med., Beyond the HIPAA Privacy Rule: Enhancing Privacy, Improving Health Through Research 66 (Sharyl J. Nass, Laura A. Levit & Lawrence O. Gostin eds., 2009) [hereinafter, ‘IOM, Privacy Report’], available at http://www.nap.edu/catalog/12458.html (reporting surveys showing ‘the majority of respondents were not comfortable with their health information being provided for research except with notice and express consent’) (Accessed Jan. 22, 2023).

99 See, eg, Ohm, *supra* note 52.

100 See discussion *supra* notes 47–52 and accompanying text.

101 See, eg, Matthew Maniaci, *Privilege, Race, and the Myth of ‘Pulling Yourself Up by Your Bootstraps,*’ Mar. 2, 2021, https://aninjusticemag.com/privilege-race-and-the-myth-of-pulling-yourself-up-by-your-bootstraps-cddf8987ee19 (arguing that privilege, luck, and circumstance play decisive roles in individual success) (Accessed Jan. 22, 2023).

102 See Balkin, *supra* note 24 at 1219 (calling for rules to “include not only robots—embodied material objects that interact with their environment—but also artificial intelligence agents and machine learning algorithms). See Pasquale, *supra* note 3, at 3, 7, 9–10, 11 (achieving similar breadth by directing his new laws of robotics at ‘robotic systems and AI’.).

103 See Eric J. Topol, *High-performance Medicine: The Convergence of Human and Artificial Intelligence*, 25 Nature Medicine 44–56 (discussing limitations of current AI/ML CDS tools, which excel at specific, narrow tasks while lacking general intelligence: for example, they can outperform human radiologists in reading chest X-rays to detect whether a patient has pneumonia, but if the answer is ‘no’, they cannot leap to the broader inquiry, ‘What else might be wrong?’).

104 See *supra* notes 7–11 and accompanying text.

105 Jon Stone, *FDA Clears AI Device for Diagnosis of Diabetic Retinopathy; Is this Favorable for Use of AI in Digital Pathology?*, Dark Daily (Jan. 2, 2019), https://www.darkdaily.com/2019/01/02/fda-clears-ai-device-for-diagnosis-of-diabetic-retinopathy-is-this-favorable-for-use-of-ai-in-digital-pathology/ (Accessed Jan. 22, 2023).

106 U.S. Gov’t Accountability Off., GAO-21-7SP, Artificial Intelligence in Health Care: Benefits and Challenges of Technologies to Augment Patient Care 21 (Nov. 2020) (highlighting these and three other challenges: difficulties in scaling software systems to serve large populations in diverse healthcare settings, ‘limited transparency of AI tools’, and ‘uncertainty about liability’).

107 See Balkin, *supra* note 24, at 1233 (discussing ‘socially unjustified use of computational capacities that externalizes costs onto innocent others’).

108
*Id*. at 1232.

109
*Id.* at 1227.

110
*Id.*

111 See Lawrence Lessig, Code and Other Laws of Cyberspace 152 (1999) (‘No one spends money collecting these data to actually learn anything about you. They want to learn about people like you.’).

112 See, eg, U.S. Gov’t Accountability Off., *supra* note 106, at 24.

113 James J. Heckman, *Selection Bias*, in Encyclopedia of Social Measurement (2005), https//www.sciencedirect.com/topics/medicine-and-dentistry/selection-bias (explaining that selection bias occurs when the group of people included in (selected for) a study are not representative of the entire population that ultimately will rely on results from that study) (Accessed Jan. 22, 2023).

114
*For concerns with racial biases in AI/ML datasets and algorithms,* see, eg, Adewole S. Adamson & Avery Smith, *Machine Learning and Health Care Disparities in Dermatology*, 154 JAMA Dermatology 1247 (2018); Ruha Benjamin, *Assessing Risk, Automating Racism*, 366 Science 421–22 (2019); Ziad Obermeyer et al*.*, *Dissecting Racial Bias in an Algorithm Used to Manage the Health of Populations*, 366 Science 447–53 (2019); Alice B. Popejoy et al*., The Clinical Imperative for Inclusivity: Race, Ethnicity, and Ancestry (REA) in Genomics*, 39 Human Mutation 1713–20 (2018). *For concerns with gender disparities,* see, eg, Carolyn Criado Perez, Invisible Women: Data Bias in a World Designed for Men (2019).

115 See, eg, Curtis S. Tenney et al., *A Crisis of Erasure: Transgender and Gender-Nonconforming Populations Navigating Breast Cancer Health Information*, International J. Information, Diversity, & Inclusion (2021) (noting that common health information norms and research practices fail to recognize all patients); Sari L. Reisner et al., *‘Counting’ Transgender and Gender-Nonconforming Adults in Health Research Recommendations from the Gender Identity in US Surveillance Group*, 2 TSQ: Transgender Studies Quarterly 34–56 (2015) (providing recommendations). See also Christine Labuski & Colton Keo-Meier, *The (Mis)Measure of Trans*, 2 TSQ: Transgender Studies Quarterly 13–33 (2015) (noting ‘transgender’s instability as a research variable’ and calling for ‘more precise methodological orientations in trans research, particularly regarding gender and sexual orientation’); T. Benjamin Singer, *The Profusion of Things: The ‘Transgender Matrix’ and Demographic Imaginaries in US Public Health*, 2 TSQ: Transgender Studies Quarterly 58–76 (2015) (cautioning that ‘[d]emographic categories are double-edged swords in that they are necessary for the redirection of resources toward socially marginalized people; at the same time, they often constitute the conditions of containment of these same people’).

116 See Zaria Gorvett, *Why Transgender People Are Ignored by Modern Medicine*, BBC (Aug. 16, 2020), https://www.bbc.com/future/article/20200814-why-our-medical-systems-are-ignoring-transgender-people (noting elevated risk of aortic aneurism in transgender females) (Accessed Jan. 22, 2023); see also Keynote Address of Karl J. Surkan, *When Health Support Becomes Data Sharing: Privacy, Security, and Governance in Peer-to-Peer Social Media Health Networks*, National Cancer Institute Symposium on Personal Control of Genomic Data for Research (2019), https://www.youtube.com/watch?v=-sSJK_LEW6U&t=1531s (at 24:53 in video) (discussing health experiences of BRCA-positive transgender male patients) (Accessed Jan. 22, 2023).

117 See Nicholson Price, *Medical AI and Contextual Bias*, 33 Harv. J.L. & Tech. 66 (2019) (discussing narrow validity of AI systems developed in resource-rich contexts when implemented in lower-resource settings). See also Eric Topol, Deep Medicine: How Artificial Intelligence Can Make Healthcare Human Again (New York: Basic Books, 2019) (discussing lack of reproducibility, narrow validity, nontransparency of data, and overblown claims about the benefits of medical software); Matthew Zook et al., *10 Simple Rules for Responsible Big Data Research*, 13 PLoS Computational Biology e1005399 (2017) (identifying similar limits); Danah Boyd & Kate Crawford, *Critical Questions for Big Data*, 15 J. Information, Communication and Society 662–679 (2012) (same).

118 World Health Organization, WHO Guidance: Ethics and Governance of Artificial Intelligence for Health xi-xii (June 28, 2021) at https://www.who.int/publications/i/item/9789240029200 (Accessed Jan. 22, 2023).

119 See Tauber, *supra* note 29, Root-Wolpe, *supra* note 29, Snead, *supra* note 32.

120 Steven J. Jacobsen et al*.*, *Potential Effect of Authorization Bias on Medical Record Research*, 74 Mayo Clinic Proc. 330 (1999); Jack V. Tu et al*.*, *Impracticability of Informed Consent in the Registry of the Canadian Stroke Network*, 350 New Eng. J. Med. 1414 (2004); Steven H. Woolf et al., *Selection Bias from Requiring Patients to Give Consent to Examine Data for Health Services Research*, 9 Archives Fam. Med. 1111 (2000); Brian Buckley et al., *Selection Bias Resulting from the Requirement for Prior Consent in Observational Research: A Community Cohort of People with Ischaemic Heart Disease*, 93 Heart 1116 (2007). See *generally* IOM, Privacy Report, *supra* note 98, at 209–14 (surveying studies of consent and selection bias).

121 See, eg, Reshma Jagsi et al*., Perspectives of Patients with Cancer on the Ethics of Rapid-Learning Health Systems*, 35 J. Clin. Oncol. 2315, 2321 (2017) (citing De Vries, RG, Tomlinson T, Kim HT et al.*, Understanding the Public’s Reservations about Broad Consent and Study-by-Study Consent for Donations to a Biobank: Results of a National Survey*, 11 PLOS One e0159113 (2016)). See also Spector-Bagdady, *supra* note 40, at 2–3 (‘[T]he research community is on notice…that there are important differences in preferences by race and ethnicity’ in terms of whether people view research as important, how comfortable they feel having their data used in research and under what conditions).

122 See, eg, Mark A. Rothstein & A.B. Shoben, *Does Consent Bias Research?* 13 Am J. Bioeth. 27–37 (2013) (arguing ‘claims about the amount of consent bias are overstated.’).

123 See Miller, *supra* note 54, at 560 (‘[S]election biases…do not in themselves constitute valid ethical reasons for waiving a requirement of informed consent.’).

124 See *supra* notes 114–118.

125 See Evans, *supra* note 71 (summarizing literature on various contributors to bias).

126
*Id*.

127 See U.S. Gov’t Accountability Off., *supra* note 106.

128 See, eg, Kayte Spector-Bagdady, *supra* note 40, at 2–3, 6–8 (recounting research abuses including the Tuskegee Syphilis Study that left Black males untreated for syphilis to observe how the disease progresses when untreated, research that purposefully infected vulnerable Guatemalans with sexually transmitted diseases, and the case of Henrietta Lacks); Nanibaa’ A. Garrison, *Genomic Justice for Native Americans: Impact of the Havasupai Case on Genetic Research,* 38 Sci. Technol. Human Values 201–223 (2013) (describing unconsented secondary use of blood specimens collected from Havasupai Tribe members); H.K. Beecher, *Ethics and Clinical Research*, 274 New Engl. J. Med. 1354–1360 (1966) (detailing research abuses against elderly, vulnerable, and cognitively impaired persons).

129 See Cynthia V. Ward, *On Difference and Equality*, 3 Legal Theory 65 and see *id*. at 65 n.1, n.2 (1997) (citing literature advancing this ‘sameness’ critique of legal liberalism since 1989).

130 Dobbs v. Jackson Women’s Health Organization, No. 19–1392, 597 U.S. ___ (2022).

131 See generally, Catherine Myser, *White Normativity in U.S. Bioethics: A Call and Method for More Pluralist and Democratic Standards and Policies*, in Defining Values and Obligations 239–258 (2007) at https://www.researchgate.net/publication/289757406_White_normativity_in_US_Bioethics_A_call_and_method_for_more_pluralist_and_democratic_standards_and_policies (Accessed Jan. 22, 2023). See also, Tsosie et al*., supra* note 50 (discussing the problematic nature of individual consent norms as applied within small tribal populations).

132 See, eg, Jensen, *supra* note 82.

133 Paul Appelbaum et al*.,**False Hopes and Best Data: Consent to Research and the Therapeutic Misconception,* 12 Hastings Center Report 20 (1987). See PPSC Report, *supra* note 41, at 567 (noting that ‘research and statistical activities generally do not lead to an immediate or direct benefit for the individual subject as such. The researcher asks for the individual’s participation or for information about him, but society as a whole, rather than the individual, is the ultimate beneficiary.’).

134 See *supra* notes 111–118 and accompanying text.

135 See Janet Woodcock & Lawrence J. Lesko, *Pharmacogenetics—Tailoring Treatments to the Outliers,* 360 NEJM 811, 811 (2009) (quoting Osler). See, eg, Niti R. Aggarwal et al*., Sex Differences in Ischemic Heart Disease: Advances, Obstacles, and Next Steps*, Circ.: Cardiovasc. Qual. & Outcomes (Feb. 15, 2018), https://pubmed.ncbi.nlm.nih.gov/29449443/) (discussing underrepresentation of women in clinical trials) (Accessed Jan. 22, 2023).

136 See *supra* notes 114–118 (citing various recent studies of selection bias).

137 Woodcock & Lesko, *supra* note 135, at 811.

138 See Evans, *supra* note 71 (recommending state-law reforms to require stricter-than-HIPAA safeguards for unconsented disclosures of PHI under the Privacy Rule).

139 Schwartz, *supra* note 38, at 820 (stating control-over-information theory is the leading paradigm). See Terry, *supra* note 67, at 146 (noting the reality of ‘health-care privacy exceptionalism’). But see Blueprint, *supra* note 1 at 5, 26–27 (calling for a notice-and-consent privacy scheme for AI systems).

140 See Blueprint, *supra* note 1, at 15–22.

141
*Id.* at 23–29.

142
*Id.* at 40–45.

143
*Id.* at 46–48.

144 See Brian Buckley et al*., Selection Bias Resulting from the Requirement for Prior Consent in Observational Research: A Community Cohort of People with Ischaemic Heart Disease*, 93 Heart 1116 (2007); IOM, Privacy Report, *supra* note 98, at 209–14 (surveying studies of consent and selection bias).

145 See Blueprint, *supra* note 1, at 30.

146
*Id*. at 9.

147 H.R. 8152 (117th Congress, 2nd Session, introduced June 21, 2022), https://www.congress.gov/117/bills/hr8152/BILLS-117hr8152ih.xml (Accessed Jan. 22, 2023).

148
*Id.* § 404.

149 See discussion *supra* notes 47–52 and accompanying text.

150
Pasquale, *supra* note 3, at 12–13. *See also id*. at 3, 7, 9–10, and 11 (stating laws for robots).

151
*Id.* at 3.

152
*Id.* at 7.

153
*Id.* at 9–10.

154
*Id.* at 11.

155
*Id.*

156 I. Glenn Cohen et al*., The Legal and Ethical Concerns that Arise from Using Complex Predictive Analytics in Health Care*, 33 Health Affairs 1139, 1139–40 (2014).

157 See Duhigg, *supra* note 21 (discussing the use of consumer data to draw health inferences).

158 See Blueprint, *supra* note 1, at 30.

159 See Ronald H. Aday & Jennifer J. Krabill, *Aging Offenders in the Criminal Justice System*, 7 Marquette’s Elder Advisor 237, 248 (2006) (noting the constitutional mandate to provide health care to imprisoned persons, which presents ‘prison health care providers with a dilemma to provide adequate treatment as they find themselves caught between the rising health needs of aging inmates and soaring health care costs.’).

160 See Food, Drug, and Cosmetic Act, Pub. L. No. 75–717, 52 Stat. 1040 (codified as amended at 21 U.S.C. §§ 1–2252), 21 U.S.C. § 321(g)(1) (subjecting articles to FDA’s drug regulations if they are ‘intended for use in the diagnosis, cure, mitigation, treatment, or prevention of disease’ or ‘intended to affect the structure or any function of the body’) and *id.* § 321(h) (incorporating similar intent provisions into the definition of an FDA-regulated medical device). See Clinical Laboratory Improvement Amendments of 1988, Pub. L. No. 100–578, 102 Stat. 2903 (codified as amended at 42 U.S.C. § 263a (2012)), 42 U.S.C. § 263a(a) (subjecting laboratories to CLIA regulation if they test biospecimens ‘for the purpose of providing information for the diagnosis, prevention, or treatment of any disease or impairment of, or the assessment of the health of, human beings’).

161 21 U.S.C. §§ 201.128, 801.4 (describing the algorithm FDA follows, and listing the types of data FDA considers, when inferring whether a drug or a device is ‘intended’ for clinical use).

162 See Duhigg, *supra* note 21 (describing results of a Target™ store’s data analysis).

163 Charlie Warzel, *All Your Data Is Health Data—And Big Tech Has It All*, New York Times (Aug. 13, 2019) (‘If the data we constantly shed is truly one of the best ways to understand our bodies…then why shouldn’t we treat it the same way we treat an EKG [electrocardiogram]?’).

164
Inst. of Med., Health Data in the Information Age: Use, Disclosure, and Privacy 191 (Molla S. Donaldson & Kathless N. Lohr, eds., 1994) (calling for legislation that protects the confidentiality of identifiable data based on ‘the data elements themselves, regardless of who holds the data.’).

165 Solove, *supra* note 31, at 1135–37.

166 U.S. Dept. of Health & Human Servs., Off. of the Secretary, CLIA Program and HIPAA Privacy Rule; Patients’ Access to Test Reports, Final Rule, 72 Fed. Reg. 7290, 7295 (Feb. 6, 2014).

167 IOM, Privacy Report, *supra* note 98.

168 See *supra* note 160 (describing statutory limits on FDA and CLIA jurisdiction). See also Food & Drug Admin. (Sept. 2022), *supra* note 11, at 15–20 (clarifying, in guidance, the distinction between FDA-regulable CDS software and other ‘non-device’ software not subject to FDA regulation).

169 See, eg, Kobbi Nissim et al*., Bridging the Gap Between Computer Science and Legal Approaches to Privacy*, 31 Harv. J. L. & Technol. 687, 707 (2018) (‘Privacy law in the United States takes a sectoral approach.’); Daniel Solove, *The Growing Problems With the Sectoral Approach to Privacy Law*, Privacy, +Security Blog, Nov. 13, 2015, https://teachprivacy.com/problems-sectoral-approach-privacy-law/ (criticizing the U.S. sectoral approach) (Accessed Jan. 22, 2023). *Cf.* Terry, *supra* note 67, at 149–151, 168–173 (characterizing medical privacy law as sectoral but describing some of the rationales for its exceptionalism).

170 See Stead, *supra* note 68; see *supra* note 56 (listing the Privacy Rule’s 27 norms allowing data disclosures).

171 See discussion *supra* notes 73–74 and accompanying text (noting that GDPR allows Member States to deviate from its general consent norm in their medical privacy laws for clinical health care data).

172 See GDPR, *supra* note 72, Art. 9.1 (according ‘special’ status to ‘personal data revealing racial or ethnic origin, political opinions, religious or philosophical beliefs, or trade union membership, and the processing of genetic data, biometric data for the purpose of uniquely identifying a natural person, data concerning health or data concerning a natural person’s sex life or sexual orientation’ and prohibiting processing of such data unless individual consent has been granted or other special circumstances apply *id.at Art. 9.2*).

173
*Id*.

174 See 42 U.S.C. § 1320d(4) (reflecting the original 1996 HIPAA statute’s definition of ‘health information’ as ‘any information, whether oral or recorded in any form or medium, that: (A) is created or received by a health care provider, health plan, public health authority, employer, life insurer, school or university, or health care clearinghouse; and (B) relates to the past, present, or future physical or mental health or condition of an individual, the provision of health care to an individual, or the past, present, or future payment for the provision of health care to an individual’). See also Genetic Information Nondiscrimination Act of 2008 § 105(a), 42 U.S.C. § 1320d-9(a) (expanding the definition of ‘health information’ that HIPAA protects to include genetic information). See also 42 U.S.C. § 1320d-9(b)(1) (stating, in a new section added by GINA, that Congress deems ‘genetic information,’ as broadly defined by GINA at 42 U.S.C. § 300gg-91, to be health information for purposes of making it subject to HIPAA’s privacy protections).

175 See *id.*

176
*Id*.

177 See 45 C.F.R. § 164.501 (defining psychotherapy notes); *id*. §§ 164.508(a)(2), 164.524(a)(1)(ii)) (restricting use and disclosure of, and individual access to, such notes). This exception is somewhat reminiscent of the Fifth Amendment privilege against self-incrimination, covering testimonial information only, but not genetic information or blood alcohol tests. The HIPAA exception for psychotherapy notes protects notes from talk therapy. Data showing that a doctor prescribed strong anti-psychotic medication or that a patient scored poorly on a cognitive test do not qualify as psychotherapy notes *id. § 164.501*.

178 U.S. Dept. of Health & Human Services, Office of the Secretary, CLIA Program and HIPAA Privacy Rule; Patients’ Access to Test Reports, Final Rule, 72 Fed. Reg. 7290, 7295 (Feb. 6, 2014).

179
*Id.* at 7296.

180 See Georgia Lowe et al*.*, *How Should We Deal with Misattributed Paternity? A Survey of Law Public Attitudes*, 8 AJOB Empirical Bioethics 234, 234 (2017), https://www.tandfonline.com/doi/pdf/10.1080/23294515.2017.1378751?needAccess=true [https://perma.cc/2N6S-JVNS] (discussing misattributed paternity, where the assumed father is not the biological father) (Accessed Jan. 22, 2023).

181 See discussion *supra* notes 73–74 and accompanying text.

182 See Solove, *supra* note 31, at 1135–37 (discussing the evolution over time of privacy norms about the human body). See also, eg, *Rochin v California*, 342 U.S. 165, 174 (1952) (characterizing unauthorized invasions of the human body as ‘offensive to human dignity’ and *Union Pac. Ry. Co. v Botsford*, 141 U.S. 250, 251 (1891) (crediting the ‘the right of every individual to the possession and control of his own person, free from all restraint or interference of others’).

183 See Charles Taylor, Sources of the Self: The Making of the Modern Identity 5 (1992) (criticizing the tendency of modern discourse to banish ontological accounts of human worth from the discussion of morality) and see *id*. at 8 (‘Ontological accounts have the status of articulations of our moral instincts. …If you want to discriminate more finely what it is about human beings that makes them worthy of respect, you have to call to mind what it is to feel the claim of human suffering, or what is repugnant about injustice, or the awe you feel at the fact of human life.’).

184
*Id.* at 13, 211–304.

185
Carl E. Schneider, The Practice of Autonomy 28 (1998).

186 See C. Hertzlich & J Pierret J, *From Self-Help to the Duty to be Healthy*, *in*Illness and Self in Society (1987) (reporting interviews conducted at a fitness center).

187 Julie Cohen, *Privacy, Visibility, Transparency, and Exposure*, 75 U. Chi. L. Rev. 181, 181–82, 186–87 (2008).

188
*Id.* See also, Julie Cohen, *Examined Lives: Informational Privacy and the Subject as Object*, 52 Stan. L. Rev. 1373, 1424–25 (2000), Schwartz, *supra* note 38, at 856–7.

189 See Daniel Solove, The Digital Person: Technology and Privacy Law in the Information Age, 97–101 (2004).

190
Michel Foucault, Discipline and Punish: The Birth of the Prison 223 (Alan Sheridan *trans*., 1977).

191 Cohen, *supra* note 187, at 186–7 (discussing insights of Kevin D. Haggerty and Richard V. Ericson, *The Surveillant Assemblage*, 51 Brit. J. Sociology 605, 615–617 (2000) and Gilles Deleuze & Felix Guattary, A Thousand Plateaus: Capitalism and Schizophrenia (Brian Massumi *trans*., 1987)).

192 Cohen, *supra* note 187, at 187.

193 See Neeraj K. Arora, *Interacting with Cancer Patients: The Significance of Physicians’ Communication Behavior*, 50 Social Science & Medicine 794 (2003) (noting that ‘patient preferences may more likely be states than traits’).

194 Cohen, *supra* note 187, at 187.

195 See Walt Whitman, Song of Myself 51, https://poets.org/poem/song-myself-51.

196 Cohen, *supra* note 187, at 187.

197 See Harlan M. Krumholz et al*., What Have We Learnt from Vioxx?* 334 BMJ 120 (2007) (discussing cardiovascular adverse events associated with Rofecoxib).

198 See Common Rule, 45 C.F.R. §46.102(l) (‘Research means a systematic investigation, including research development, testing, and evaluation, designed to develop or contribute to generalizable knowledge.’). See HIPAA Privacy Rule, 45 C.F.R.§ 164.501 (defining research as ‘a systematic investigation, including research development, testing, and evaluation, designed to develop or contribute to generalizable knowledge’).

199 See Michigan Medicine, University of Michigan, Munchausen Syndrome by Proxy, https://www.uofmhealth.org/health-library/hw180537#:~:text=Munchausen%20syndrome%20by%20proxy%20(MSBP,child%20abuse%20or%20elder%20abuse (‘Munchausen syndrome by proxy (MSBP) is a mental health problem in which a caregiver makes up or causes an illness or injury in a person under his or her care, such as a child, an elderly adult, or a person who has a disability.’) (Accessed Jan. 22, 2023).

200 See Foucault, *supra* note 190.

201 See *supra* note 5; Barth et al*.*, *supra* note 5 (summarizing Nissenbaum’s concept of privacy as contextual integrity).

202
*Id*. at 185.

203
*Id*. at 186.

204
*Id.*

205
*Id.* at 185–86.

206
*Id.*

207 Balkin, *supra* note 61*, at* 1187, 1205.

208 See Terry, *supra* note 67, at 197 (noting that ‘patients have been conditioned to disclose all data to their healthcare providers on the basis of this very promise; that such data will be protected like no other’).

209 Barth et al*., supra* note 5, at 186.

210
*Id.*

211
*Id.* (noting that it is largely in relation to the ends and values of a contexts that ‘the roles and norms of a context make sense’).

212 See Snead, *supra* note 32, at 70–71.

213 See *supra* notes 29, 32.

214 See Terry, *supra* note 67, at 202–203 (suggesting that stronger protections may be warranted because of the lower fiduciary duties of non-clinical data handlers).

215 See *supra* notes 113–118 and accompanying text.

216 See Stead, *supra* note 68; HHS, 1997 Recommendations, *supra* note 75.

217 45 C.F.R. § 164.524 (HIPAA access right); see also American Health Lawyers Association, 1 Health L. Prac. Guide § 4:11 (2022) (noting that, under the medical records laws of most states, patients have a right to review their medical records).

218 45 C.F.R. § 164.524.

219 See generally Sandra Wachter & Brent Mittelstadt, *A Right to Reasonable Inferences: Re-Thinking Data Protection Law in the Age of Big Data and AI*, 2019 Columbia Bus. L. Rev. 494 (2019) (discussing how ‘individuals are granted little control or oversight over how their personal data is used to draw inferences about them’ under GDPR and noting, ‘Data subjects’ rights to know about (Articles 13–15), rectify (Article 16), delete (Article 17), object to (Article 21), or port (Article 20) personal data are significantly curtailed for inferences.’ *id.* at 494–95).

220
*Id.*

221 See Carol M. Rose, *Possession as the Origin of Property*, 52 U. CHI. L. REV. 73 (1985) (describing Locke’s theory that individuals establish ownership of a thing by commingling their labor with it, and citing Lawrence Becker, Property Rights: Philosophic Foundations 49 (1977) for the notion that a psychological sense of desert is a driving force behind the appeal of this labor theory).

222 See Furrow et al., *supra* note 87, at 117.

223
Lessig, *supra* note 111, at 152.

224 See HHS, 1997 Recommendations, *supra* note 75 (designating these flows as ‘national priorities’).

225 See Barbara J. Evans, *Much Ado About Data Ownership*, 25 Harv. J. L. & Tech., 69, 226 (2011).

226
Charles Taylor, Philosophy and the Human Sciences, 2 Philosophical Papers 218 (1985).

227
Emilio Santoro, Autonomy, Freedom and Rights: A Critique of Liberal Subjectivity 247 (2003).

228
Taylor, *supra* note 226.

229 Barth et al*., supra* note 5, at 186.

230 See Evans, *supra* note 71 (discussing needed reforms).

